# A familial case of FLAD1 protein deficiency associated with impaired adrenal steroidogenesis

**DOI:** 10.1172/jci.insight.198799

**Published:** 2026-09-08

**Authors:** Olga A. Averina, Natalia Yu. Kalinchenko, Vitaly A. Ioutsi, Andrey V. Pirogov, Alexander V. Bogachev, Oleg A. Permyakov, Vitaly S. Buev, Ekaterina A. Guseva, Anastasia V. Priymak, Olga A. Bazhanova, Mariia A. Emelianova, Olga O. Grigoryeva, Galina V. Baydakova, Maxim A. Abakumov, Vasily N. Manskikh, Olga A. Dontsova, Petr V. Sergiev, Anatoly N. Tiulpakov

**Affiliations:** 1 Belozersky Institute of Physico-Chemical Biology, Lomonosov Moscow State University, Moscow, Russia.; 2 Endocrinology Research Centre, Moscow, Russia.; 3 Faculty of Chemistry, Lomonosov Moscow State University, Moscow, Russia.; 4 Center for Bio- and Medical Technologies, Skolkovo Institute of Science and Technology, Skolkovo, Russia.; 5 Research Centre for Medical Genetics, Moscow, Russia.; 6 Pirogov Russian National Research Medical University, Moscow, Russia.; 7 Shemyakin–Ovchinnikov Institute of Bioorganic Chemistry, Moscow, Russia.

**Keywords:** Endocrinology, Genetics, Metabolism, Bioenergetics, Genetic diseases, Mouse models

## Abstract

The *FLAD1* gene codes for flavin adenine dinucleotide (FAD) synthase. FAD is a cofactor for many redox enzymes involved in vital processes from respiration to signal transduction. In this work, we described a clinical case of 2 siblings carrying compound heterozygous mutations in the *FLAD1* gene resulting in the substitutions A418V and R542* at the protein level. The patients demonstrate adrenal insufficiency, which has not previously been associated with FLAD1 protein defects. To verify that adrenal insufficiency is caused by *FLAD1* mutations, we created a personalized mouse model carrying the mutations found in the patients. The mutation in the *FLAD1* gene, leading to the A418V substitution, appeared viable in the homozygous state, with minimal difference from the WT. The *FLAD1* gene mutation leading to the R542* truncation is lethal when homozygous. The mouse model of the compound heterozygous *FLAD1*^A418V/R542*^ mutations recapitulated the physiological, biochemical, and endocrine manifestations of *FLAD1* mutations in patients. The mouse model created demonstrates the causal effect of FLAD1 mutations on the described pathology and potentially paves the way for understanding the disease’s molecular mechanism and developing better therapies.

## Abstract 2

Novel variants of FLAD1 gene were found to cause adrenal insufficiency in humans and mice

## Introduction

In humans, several steroidogenic steps require cytochrome P450 enzymes for cortisol biosynthesis. Deficiencies of these enzymes, namely CYP11A1, CYP17A1, CYP21A2, and CYP11B1, comprise the 4 well-characterized forms of monogenic autosomal recessive disorders grouped under the term of congenital adrenal hyperplasia (CAH). Both microsomal and mitochondrial P450 enzymes, including those involved in steroidogenesis, receive 2 electrons from NADPH transferred via flavoproteins. P450 oxidoreductase (POR), containing FAD and FMN, transfers electrons to PORs of the endoplasmic reticulum, while FAD-containing ferredoxin reductase (FDXR) transfers electrons to mitochondrial P450 enzymes via ferredoxin (FDX).

To date, at least 2 monogenic defects in cortisol biosynthesis have been described, resulting from deficiencies in one of the P450 partner proteins that ensure electron transfer. One form of CAH is caused by a deficiency of POR, a flavoprotein that donates electrons to microsomal P450 enzymes, CYP17A1 and CYP21A2 (1, 2). Recently, Pignatti and coauthors (3) described a form of adrenal insufficiency caused by a defect in FDXR, a flavoprotein that mediates electron transfer to mitochondrial P450 enzymes, such as CYP11A1 and CYP11B1 (3). Since the redox partners cooperate with different cytochrome P450 enzymes throughout the body, their deficiency may cause a broad spectrum of clinical symptoms, not limited to changes in steroid hormones, and may even be present without them. POR deficiency, for instance, may manifest with skeletal malformations, attributed to the concomitant impairment of CYP26B1 activity (4). FDXR deficiency is characterized by generalized mitochondriopathy with a variable clinical spectrum and severity (4), and the familial case described by Pignatti et al. to date represents the only example of this disorder to our knowledge in which mitochondrial neuropathy coincided with impaired steroidogenesis (CYP11B1 deficiency) (3).

Flavin adenine dinucleotide (FAD) is a ubiquitous component of a multitude of redox enzymes indispensable for energy metabolism as well as for many other processes. In total, the human genome encodes 90 flavin-dependent enzymes, 84% of which contain FAD (5). Dietary sources, mainly milk and eggs, supply free riboflavin (vitamin B2) and FAD/FMN bound to proteins. Flavoprotein denaturation and fragmentation in the stomach, followed by the activity of phosphatases and pyrophosphatases in the ileum, releases additional free riboflavin. SLC52A3 transporter is responsible for riboflavin uptake in the small intestine, while SLC52A1 and SLC52A2 release riboflavin or FMN into the bloodstream. SLC52A2 is also responsible for riboflavin transport into other cell types. An additional protein, SLC25A32, transports FAD across the inner mitochondrial membrane. Excess of riboflavin, which has not been converted to FAD and FMN or bound to riboflavin-binding proteins, is rapidly excreted by the kidneys.

**Figure 1:**
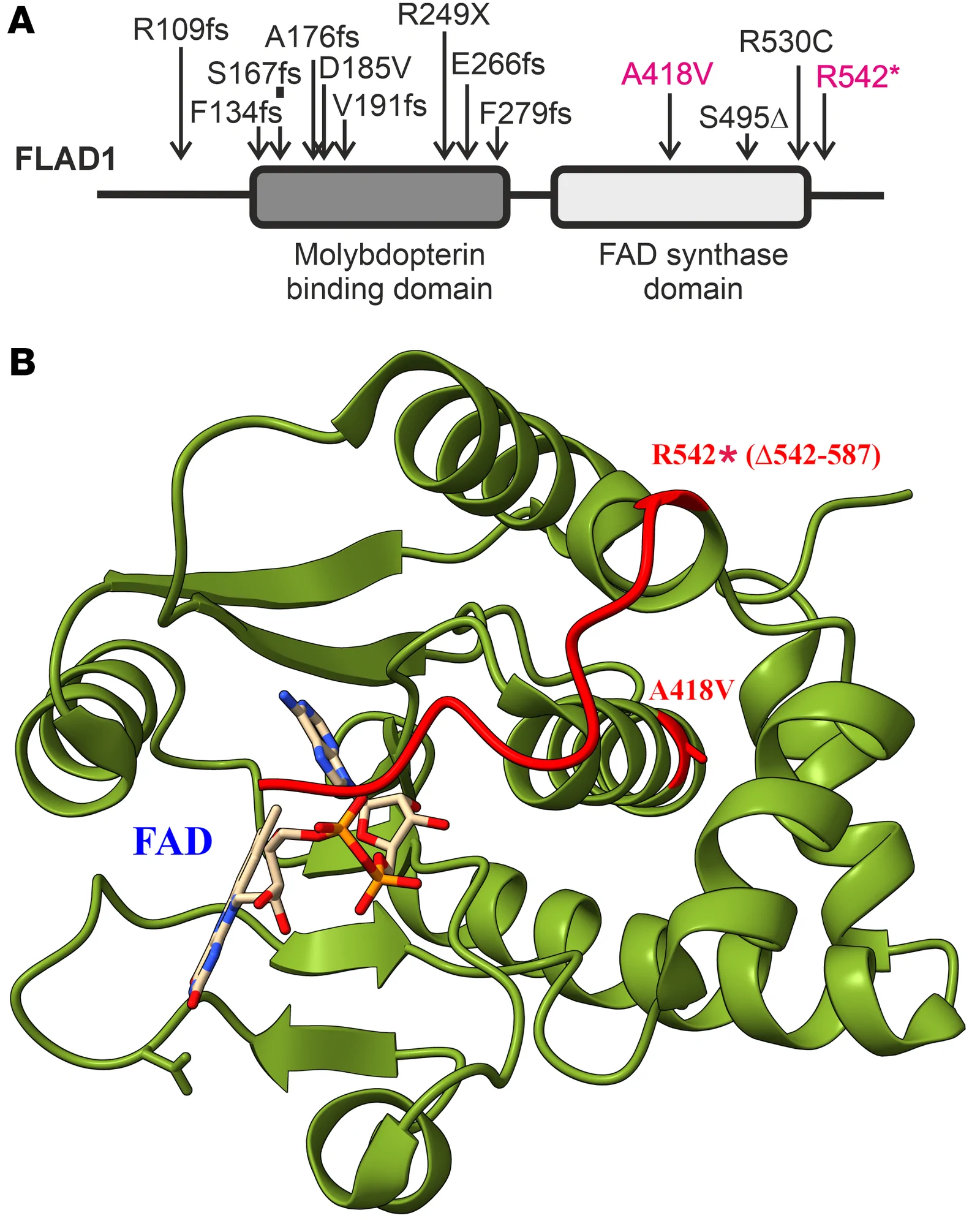
Structure of the FAD synthase with the variants marked. (**A**) Domain organization of the FLAD1 protein. The molibdopterin binding domain and FAD synthase domain are shown and marked. Known pathogenic variant sites are indicated by arrows and labeled accordingly. The variants described herein are marked by pink. (**B**) Structure of the FAD synthase domain (28) of the human FLAD1 protein (PDB:8rom). The FAD molecule is shown as a wireframe model colored CPK. The residue changed by the A418V variant and the fragment absent in the R542* mutant are colored red.

FAD is synthesized by a single protein, FLAD1, in mammals (6). Upstream from FLAD1, riboflavin is converted to flavin mononucleotide (FMN) by the riboflavin kinase (RFK). The *FLAD1* gene is localized on chromosome 1 at 1q21.3. Two splicing isoforms are studied: one mitochondrial and the other cytosolic (7, 8). FLAD1 protein contains an N-terminal molibdopterin binding domain and a C-terminal FAD synthase (ATP:FMN adenylyl transferase) domain (Figure 1A). The former is hypothesized to hydrolyze FAD under yet-to-be-defined conditions (9). After FAD synthesis, the product remains tightly bound to FLAD1 until it is released to one of a multitude of apoflavoproteins, suggesting FAD chaperone activity (10, 11). Another shorter FLAD1 isoform, named hFADS6, has been characterized in patients carrying frameshift variants in the N-terminal molybdopterin-binding domain (12). This isoform is suggested to compensate for severe FLAD1 deficiency upon these variants. Interestingly, 7 frameshift variants with clinical manifestations reported in the literature are found exclusively in the N-terminal FLAD1 molibdopterin binding domain, while missense and in-frame deletion have been reported in the FAD synthase C-terminal part (13) (Figure 1A).

Multiple acyl-coenzyme A dehydrogenation deficiency (multiple acyl-CoA dehydrogenase deficiency [MADD], MIM #231680) is an autosomal recessive disorder that affects the oxidation of fatty acids, several amino acids, and choline. Known variants leading to MADD are found in genes encoding electron transfer flavoprotein dehydrogenase (ETFDH), the α (ETFA) and β (ETFB) subunits of electron transfer flavoprotein; the riboflavin transporter genes SLC52A1, SLC52A2, and SLC52A3; and the mitochondrial FAD transporter gene *SLC25A32*. MADD-like symptoms may also be caused by variants in the *FLAD1* gene (6). Out of a total of > 400 variants described as causative for MADD, only 20 have been attributed to *FLAD1* in 2021 (14–22) (Supplemental Table 1; supplemental material available online with this article; https://doi.org/10.1172/jci.insight.198799DS1). A typical manifestation of MADD in general and FLAD1 insufficiency in particular is lipid droplet accumulation in muscle fibers, clearly visible by Oil Red O staining (13, 16, 18, 19, 23, 24) due to the reduced ability to oxidize fatty acids. The same mechanism leads to an increase in the concentration of several fatty-acid acyl carnitines in blood plasma (13, 16, 17, 19, 20, 22, 24, 25).

We report here 2 siblings (1 boy, 1 girl) with compound heterozygous A418V and R542* variants in the *FLAD1* gene, who presented with primary adrenal insufficiency, myopathy, and biochemical abnormalities consistent with MADD. Defects in the *FLAD1* gene are described in lipid storage myopathy due to FAD synthetase deficiency (MIM# 255100), a rare autosomal recessive inborn error of metabolism that includes variable mitochondrial dysfunction but, to the best of our knowledge, has not yet been associated with primary adrenal insufficiency.

Using a personalized mouse model, we present evidence that the Flad1^A418V/R542*^ genotype is associated with adrenal dysfunction, as well as other features consistent with previously described characteristics of FLAD1 deficiency in humans.

## Results

### Clinical cases.

**Figure 2:**
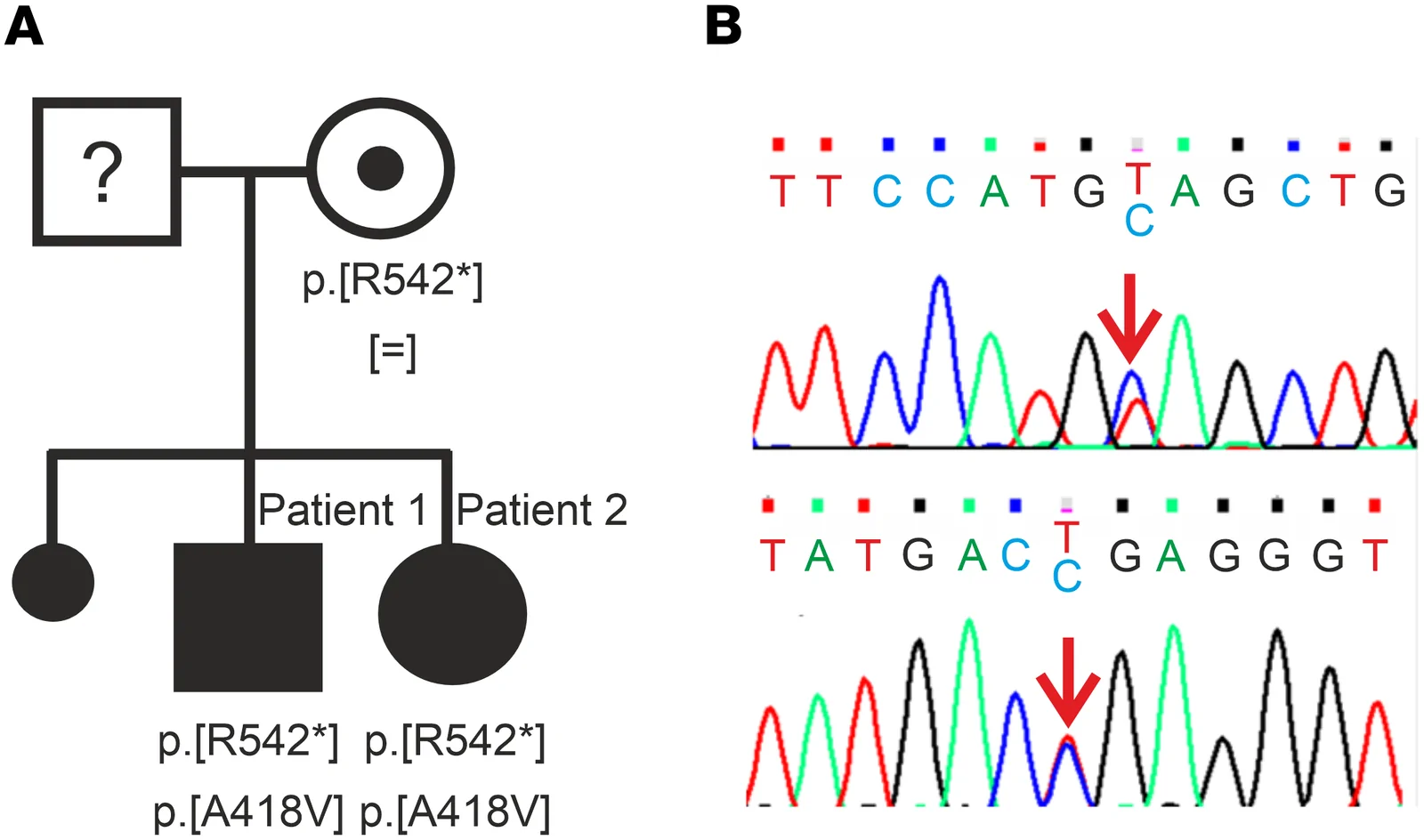
Patients’ pedigree and genotype. (**A**) Patients’ pedigree. Shown are the known data on patients’ and their relatives’ genotypes, where known. (**B**) Patient’s genotype. Shown are the results of Sanger sequencing of the amplicons of the FLAD1 gene for patient 2 (identical results were obtained for patient 1). The upper panel corresponds to the region coding for the amino acid 418, while the lower panel corresponds to that of amino acid 542. Variant sites were marked by the red arrow.

Two sibling patients, the boy (Patient 1) and the girl (Patient 2), were born to nonconsanguineous parents (Figure 2A). There was no history of endocrine or neuromuscular diseases in the family. Both patients were delivered by cesarean section due to preterm labor and fetal distress (Table 1). At the age of 17 days (Patient 1) and 6 days (Patient 2), adrenal insufficiency was diagnosed, manifested by low cortisol levels, hyponatremia, and hyperkalemia (Table 1). Replacement with hydrocortisone and fludrocortisone has been initiated. While on corticosteroid treatment, the patients repeatedly showed elevated plasma ACTH levels (Table 1). At age 4 months (Patient 1) and 6 months (Patient 2), bilateral hip dysplasia was diagnosed. Later, at ages 4–5 years, Klippel-Feil syndrome (fusion of cervical vertebrae and severe scoliosis) has been observed. Both patients suffered from bilateral severe pneumonia at an early age (<1 year) and showed muscle hypotonia. Blood glucose levels were typically within the lower normal range. Increased levels of C8, C10, and C10:1 acyl carnitines were observed in Patient 1 at ages 8.6 and in Patient 2 at 6.9 years, respectively (Table 1).

Whole exome sequencing in Patient 1 revealed 2 heterozygous variants in the *FLAD1* gene (NM_025207): c.1253C>T (p.A418V) in exon 3 and c.1624C>T (p.R542*) in exon 6; the latter one leading to the formation of an in-frame stop codon (Figure 2B). Compound heterozygosity in Patient 1 and Patient 2 was confirmed by amplification of genomic DNA fragments and Sanger sequencing. According to amplicon sequencing, the mother was heterozygous for the p.R542* variant. The father was not available for DNA analysis.

Following detection of mutations in the *FLAD1* gene, riboflavin (100 mg, daily) was administered to both patients. After 6 months of riboflavin treatment, blood levels of fatty acyl carnitines fell within normal values (Table 1).

### Potential pathogenicity of the FLAD1^A418V^ and FLAD1^R542*^ variants.

FLAD1 variants observed in patients described herein have not been previously described as pathogenic. To assess their potential pathogenicity, we compared corresponding allele frequencies and pathogenicity predictions (26, 27) with those of the previously described FLAD1 pathogenic alleles (Supplemental Table 1). Both alleles leading to A418V and R542* substitutions have been previously observed in human genome sequencing at frequencies comparable with those of other pathogenic mutations (28), although neither was found in patients (Supplemental Table 2). Pathogenicity prediction tools such as PolyPhen2 (26) and FATHMM (27) suggest A418V is possibly pathogenic or tolerable, while the residue is not absolutely conserved among FLAD1 orthologs (26). The R542* mutation introduces a premature stop codon; thus, its consequences have not been predicted.

To delve deeper into the potential pathogenicity of the A418V and R542* variants, we used available structures of the FLAD1 protein (28). The C-terminal part of FLAD1 (Figure 1B) was shown to be required for its FAD synthase activity, as the removal of the last 34 amino acids totally inactivates the protein (28), which is explained by the removal of conserved R583-R586 residues likely interacting with phosphate groups of the substrates (28). Thus, the FLAD1^R542*^ variant is likely to be inactive. Residue A418 is located in the hydrophobic core of the protein, formed by 3 α-helices (Figure 1B). It is likely that replacement of the compact alanine with the bulkier valine might destabilize FLAD1 folding, albeit not inactivate the enzyme completely, in line with the predictions (26, 27).

### Creation of a personalized mouse model carrying patients’ FLAD1 variants.

Computational predictions (Supplemental Table 1) and literature analysis (Supplemental Table 2) do not provide a clear understanding regarding the pathogenicity of FLAD1 A418V and R542* variants. Moreover, the endocrine pathology characteristic of the patients described herein has not been previously ascribed to patients carrying *FLAD1* variants. To obtain a clear-cut conclusion on whether the FLAD1^A418V/R542*^ compound heterozygous variants resulted in adrenocortical insufficiency, we established a personalized mouse model for the patients. To this end, we microinjected Cas9 mRNA, sgRNAs targeting the corresponding regions of the murine genome, and single-stranded DNA templates for homologous recombination in a separate set of experiments aimed to create independent FLAD1^A418V^ and FLAD1^R542*^ alleles. Heterozygous mouse founders carrying either *FLAD1^wt/A418V^* or FLAD1^wt/R542*^ (Supplemental Figure 1) variants have been back-crossed to the C57BL6/J background and, when mated, aim to obtain homozygous *FLAD1^A418V/A418V^* and FLAD1^R542*/R542*^ mice lines as well as a compound heterozygous FLAD1^A418V/R542*^ mice population.

**Figure 3:**
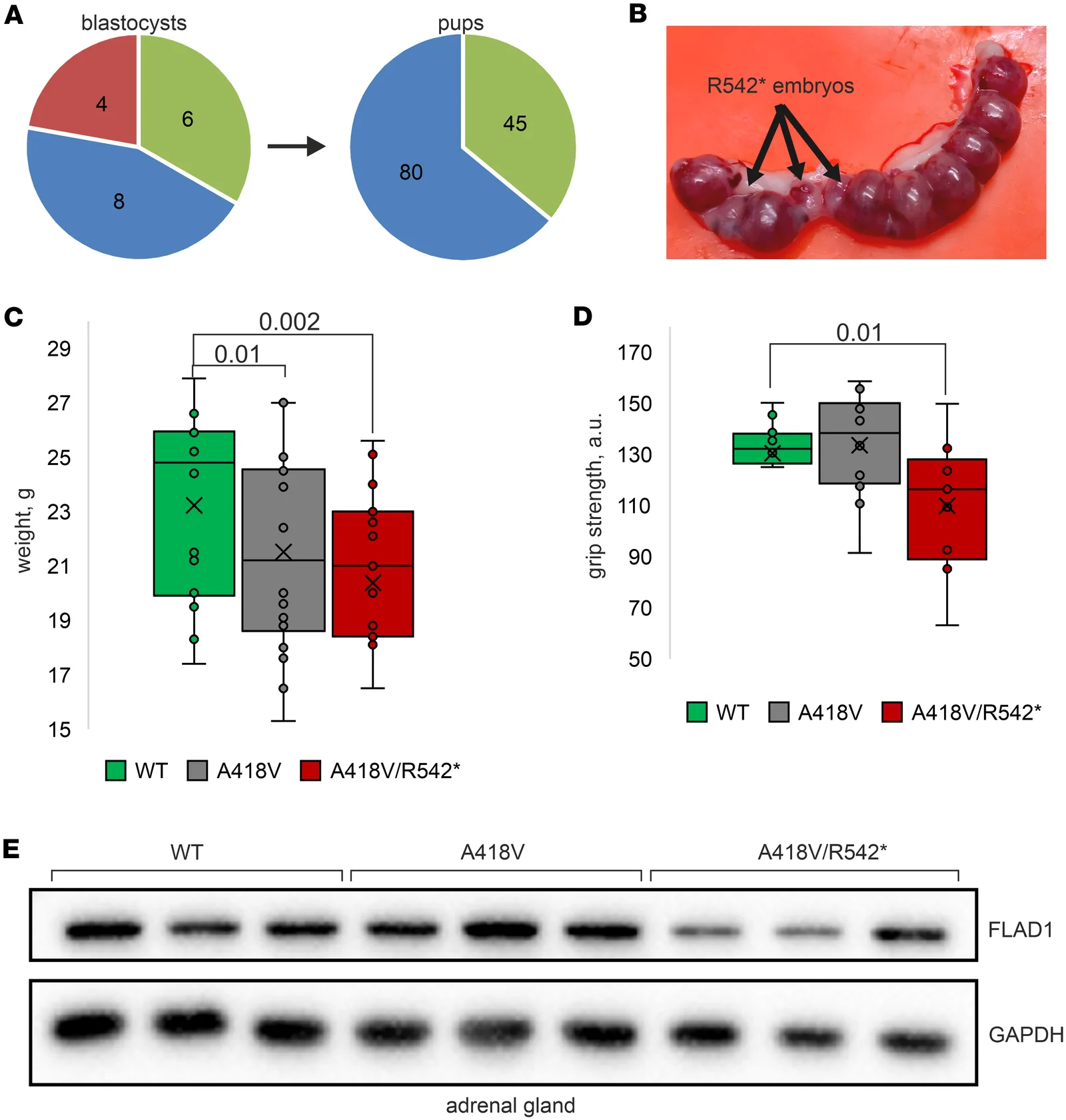
Variant *FLAD1*^R542*/R542*^ is lethal, *FLAD1*^A418V/R542*^ has a severe phenotype, and *FLAD1*^A418V/^ ^A418V^ has a moderate one. (**A**) Number of different *FLAD1* genotypes at the blastocyst stage (left panel) and at birth (right panel) after mating of heterozygous *FLAD1*^wt/R542*^ mice. Red corresponds to the homozygous mutants *FLAD1*^R542*/R542*^, blue corresponds to heterozygotes *FLAD1*^wt/R542*^, while green corresponds to the WT. (**B**) Uterus of the mice pregnant by embryos of genotype *FLAD1*^R542*/R542*^, *FLAD1*^wt/R542*^, and *FLAD1*^wt^. Embryos that were later genotyped as homozygous *FLAD1*^R542*/R542*^ are marked by arrows. (**C**) The weight of male mice (6 months old) depends on genotype. The green corresponds to the WT (*n* = 11), the gray to *FLAD1*^A418V/A418V^ (*n* = 12), and the red to *FLAD1*^A418V/R542*^ (*n* = 9) mice. *P* = 0.0057, Kruskal-Wallis statistic = 10.33 by Kruskal-Wallis test with the Benjamini-Krieger-Yekutieli correction for multiple comparisons. (**D**) Grip strength of mice (male, 6 months) is dependent on the genotype. The color code is the same as for **C** (WT *n* = 12, FLAD1^A418V/A418V^ *n* = 12, FLAD1^A418V/R542*^ *n* = 8). *P* = 0.0147, Kruskal-Wallis statistic = 8.439 by Kruskal-Wallis test with the Benjamini-Krieger-Yekutieli correction for multiple comparisons. (**E**) Immunoblotting of the adrenal gland extracts with anti-FLAD1 (upper panels) or anti-GAPDH (lower panel, loading control) antibodies. The FLAD1 genotypes of the mice analyzed are indicated above the lanes as follows: WT (*n* = 3, 4 months, male), A418V (*n* = 3, 4 months, male), and A418V/R542* (*n* = 3, 4 months, male).

Somewhat expected, *FLAD1*^A418V/A418V^ and *FLAD1*^A418V/R542*^ mice were found to be viable, while *FLAD1*^R542*/R542*^ mice were apparently lethal, as we never succeeded in obtaining pups carrying this genotype while mating *FLAD1*^wt/R542*^ mice (Figure 3A). Genotyping of embryos at the E4.5, E8.5, E11.5, and E14.5 revealed that while blastocysts of the *FLAD1*^R542*/R542*^ genotype are still viable (Figure 3A), later stage embryos of this genotype stop developing and are resorbed (Figure 3B).

The following analysis demonstrated that *FLAD1*^A418V/A418V^ mice have a mild but detectable phenotype, while a compound heterozygote *FLAD1*^A418V/R542*^ combining presumed hypofunctional and completely inactivating variants demonstrated a more severe phenotype. The weight of mice (Figure 3C) and the grip strength (Figure 3D) were most affected by the *FLAD1*^A418V/R542*^. Unlike patients reported herein and several other patients (13) with scoliosis, mice carrying even the severe *FLAD1*^A418V/R542*^ genotype do not develop scoliosis and are characterized only by a slight tendency toward decreased bone density (Supplemental Figure 2).

### The variants decrease the concentration and activity of the FLAD1 protein.

Variants introduced to the FLAD1 protein might destabilize it. To address this issue, we analyzed the concentration of FLAD1 protein in mice’s oxidative (soleus) (Supplemental Figure 3, A and D) and glycolytic (tibialis anterior) (Supplemental Figure 3, B and E) muscles, as well as in the adrenal gland (Figure 3E and Supplemental Figure 3, C and F). The latter is important since patients described herein have adrenal insufficiency. For all tissues analyzed, a compound heterozygote FLAD1^A418V/R542*^ demonstrated a lower amount of FLAD1 protein, while FLAD1^A418V/A418V^ mice demonstrated a decrease in the protein amount only for muscles. Although we do not have a straightforward explanation for this tissue and allele specificity, the general trend is that compound-heterozygous *FLAD1*^A418V/R542*^** mice exhibit the most severe phenotype across all assays. Thus, both variants seem to either destabilize the FLAD1 protein or decrease its gene expression.

**Figure 4:**
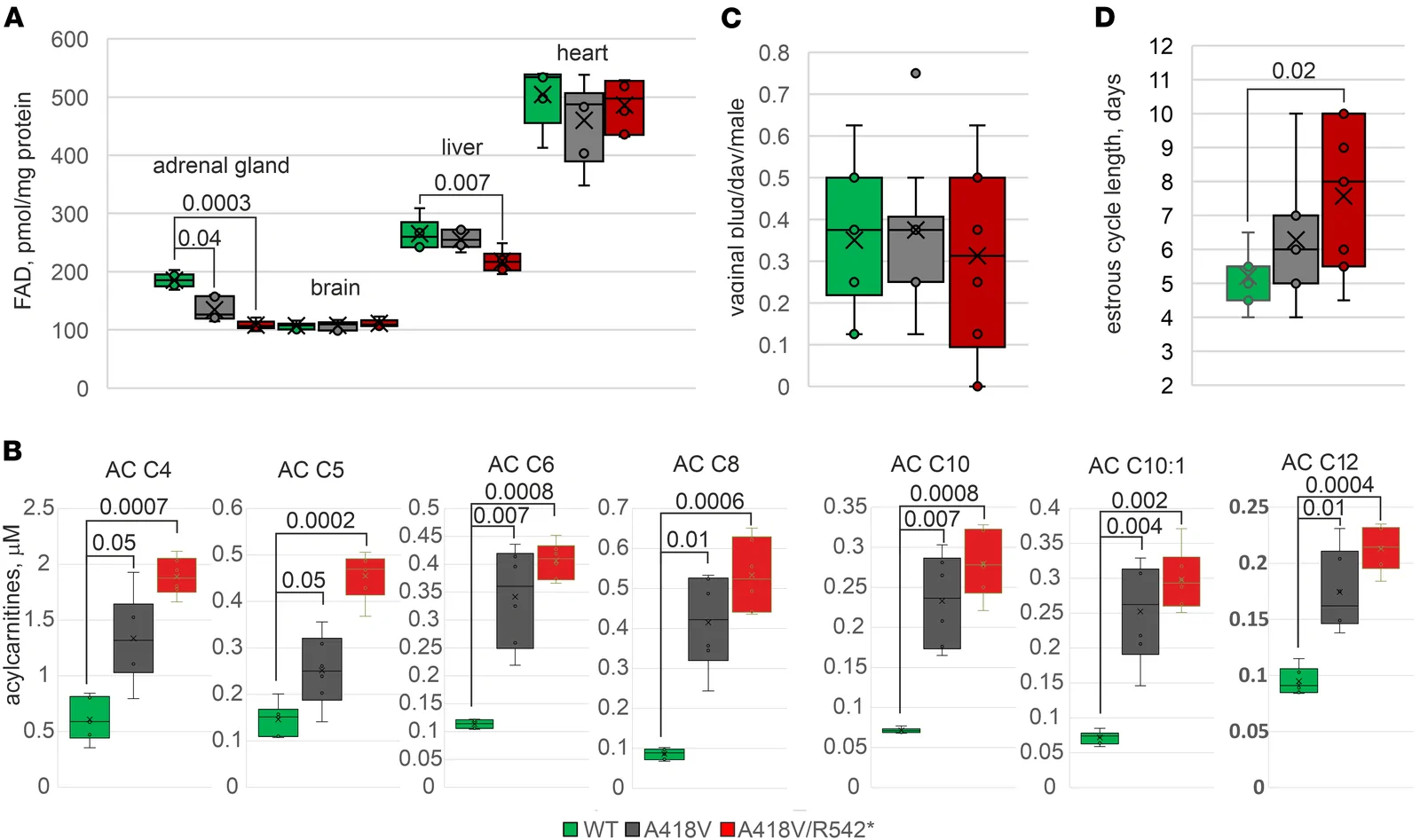
Metabolite quantity in mice with mutated *FLAD1*. (**A**) FAD content in mouse tissues, such as the adrenal gland, brain, liver, and heart extracts, as marked above the corresponding bars. Green corresponds to the WT (*n* = 6, 4 months, male), gray to the homozygous *FLAD1*^A418V/A418V^ mutation (*n* = 6, 4 months, male), and red to the compound heterozygous *FLAD1*^A418V/R542*^ mice (*n* = 6, 4 months, male). FAD concentration is normalized by protein content (BCA), pmol/mg protein. *P* < 0.0001, Kruskal-Wallis statistic = 14.58 for adrenal gland; *P* = 0.7484, Kruskal-Wallis statistic = 0.6371 for brain; *P* = 0.0061, Kruskal-Wallis statistic = 8.846 for liver; *P* = 0.2899, Kruskal-Wallis statistic = 2.575 for heart by Kruskal-Wallis test with the Benjamini-Krieger-Yekutieli correction for multiple comparisons. (**B**) Blood spot concentration of acyl carnitines (indicated above the graphs), whose concentration is most affected by *FLAD1* variants. The color code, the same as for **A**, is indicated on the graph; *n* = 6 for all groups presented (except *FLAD1*^A418V/A418V^ group in AC C4 being *n* = 5). *P* < 0.0001, Kruskal-Wallis statistic = 13.07 for AC C4; *P* < 0.0001, Kruskal-Wallis statistic = 13.66 AC C5; *P* = 0.0002, Kruskal-Wallis statistic = 12.12 for AC C6; *P* < 0.0001, Kruskal-Wallis statistic = 12.54 AC C8; *P* < 0.0001, Kruskal-Wallis statistic = 12.13 AC C10; *P* = 0.0003, Kruskal-Wallis statistic = 11.66 AC C10:1; *P* < 0.0001, Kruskal-Wallis statistic = 12.94 AC C12 by Kruskal-Wallis test with the Benjamini-Krieger-Yekutieli correction for multiple comparisons. (**C**) Frequency of male copulation as measured by the average number of vaginal plugs observed per male per day (10 days total). Green corresponds to the WT (*n* = 8, 4 months, male), gray to the homozygous *FLAD1*^A418V/A418V^ mutation (*n* = 8, 4 months, male), and red to the compound heterozygous *FLAD1*^A418V/R542*^ mice (*n* = 8, 4 months, male). No statistically significant difference was observed between the groups (χ^2^ = 1.103, df = 2, *P* = 0.576). (**D**) Estrous cycle length, days (totally 14 days measured, daily). Green color corresponds to the WT (*n* = 7, 4 months, female), gray color to the homozygous *FLAD1*^A418V/A418V^ mutation (*n* = 7, 4 months, female), and red to the compound heterozygous *FLAD1*^A418V/R542*^ mice (*n* = 7, 4 months, female). *P* = 0.0322, Kruskal-Wallis statistic = 6.516 by Kruskal-Wallis test with the Benjamini-Krieger-Yekutieli correction for multiple comparisons.

Since the primary catalytic activity of FLAD1 is FAD synthesis, we measured FAD concentration in adrenal gland, brain, liver, and heart extracts from WT and mutant mice (Figure 4A). FAD concentration was found to decrease in the adrenal glands of both *FLAD1*^A418V/R542*^ and *FLAD1*^A418V/A418V^ mice and in the liver of the compound heterozygous *FLAD1*^A418V/R542*^ mice. FAD content of other tissues appeared to be more prone to *FLAD1* mutations. In tissues where we observed a difference in FAD concentration, compound-heterozygous *FLAD1*^A418V/R542*^ mice showed a more substantial decrease in FAD concentration compared with *FLAD1*^A418V/A418V^ mice, which showed a tendency toward a decrease in FAD concentration that did not reach statistical significance.

Expectedly, no difference in riboflavin (Supplemental Figure 4A) and FMN (Supplemental Figure 4B) concentration has been detected for either mutant.

### Variants in the FLAD1 gene affect mice’s acyl carnitine profile, similar to that of patients, while having minimal effects on reproductive functions.

An increase in the concentration of several fatty acid acyl carnitines has previously been reported (13, 15–17, 19–22, 24, 25) and described herein for patients with the FLAD1 variants. We analyzed acyl carnitine profiles in the blood spots of the WT mice and mice carrying FLAD1^A418V/R542*^ and FLAD1^A418V/A418V^ genotypes by mass spectrometry (Figure 4B and Supplemental Figure 4C). We observed a profound increase in the concentrations of C4, C5, C6, C6OH, C8, C8:1, C10, 10:2, C10:1, C12, C12:1, C14:2, and C22 acyl carnitines for both mutant mouse lines. In addition, several other acyl carnitines and several amino acids demonstrated an elevation in the blood of mice carrying the FLAD1^A418V/R542*^ genotype (Supplemental Figure 4C).

Mutations in the FLAD1 gene, to a small extent, affect gonadal function. While the male ability to copulate remained indistinguishable from that of the WT for both FLAD1^A418V/R542*^ and FLAD1^A418V/A418V^ variants (Figure 4C), the female estrous cycle is delayed for the FLAD1^A418V/R542*^ mice, while that of FLAD1^A418V/A418V^ is indistinguishable from the WT (Figure 4D).

### The variants in the FLAD1 gene cause adrenal insufficiency in a mouse model.

**Figure 5:**
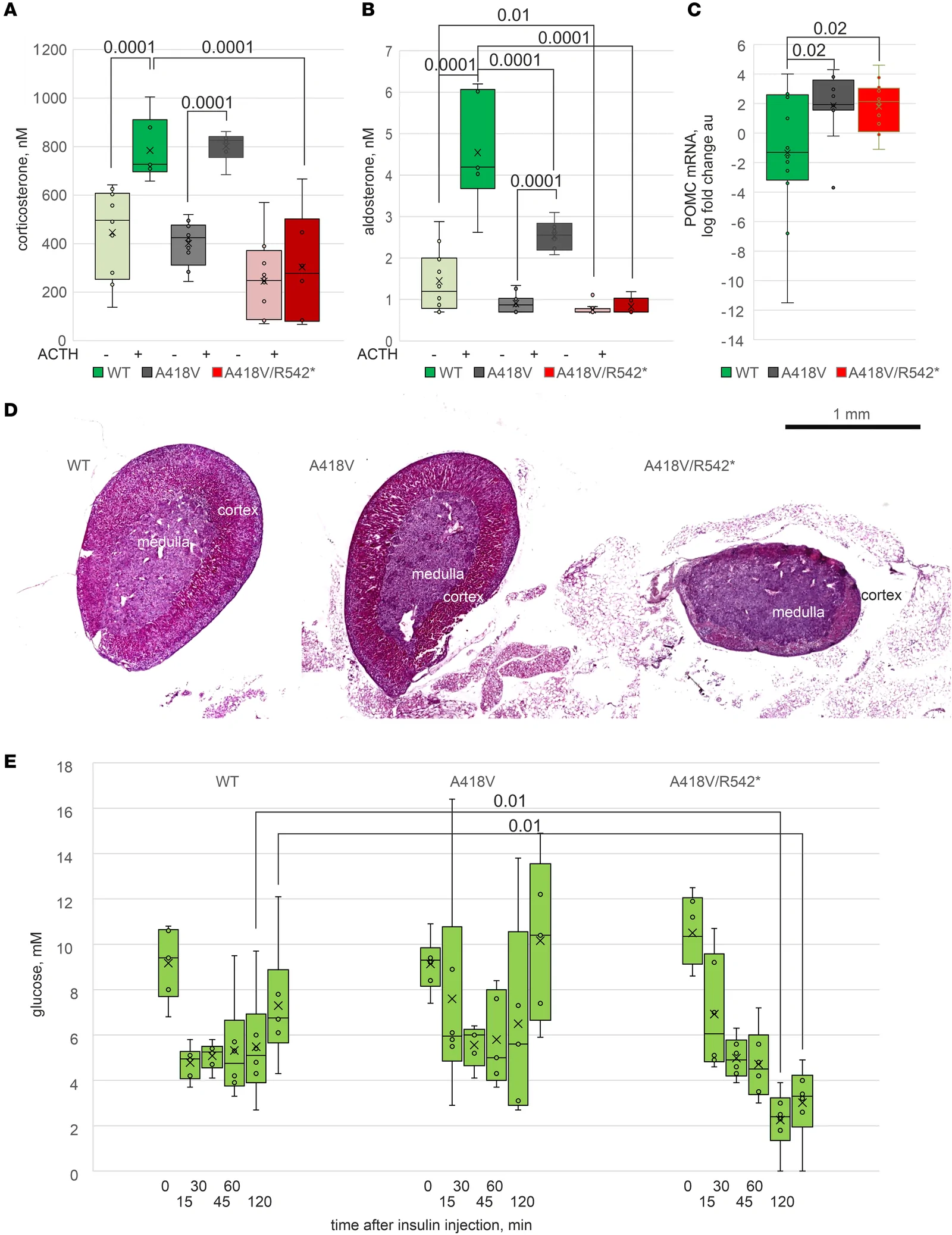
*FLAD1* variants lead to adrenal insufficiency and hypoglycemia. (**A**) Corticosterone concentration in blood plasma at steady state (pale colors, bars 1, 3, 5) and after ACTH (synacthen) stimulation (bright colors, bars 2, 4, 6); the data is presented in nM. Green corresponds to the WT (bars 1, 2, *n* = 12, 3 months, male), gray color corresponds to *FLAD1*^A418V/A418V^ (bars 3, 4, *n* = 12, 3 months, male), while red color corresponds to the *FLAD1*^A418V/R542*^ (bars 5, 6, *n* = 12, 3 months, male) mice. Statistical analysis was performed using a mixed-effects model (REML): significant effects of ACTH (synacthen) stimulation [F(1,15) = 83.50, *P* < 0.0001], Genotype [F(2,33) = 19.58, *P* < 0.0001], and ACTH×Genotype interaction [F(2,15) = 17.58, *P* = 0.0001]. Post hoc: 2-stage linear step-up procedure of Benjmini, Krieger, and Yekutieli (5 families, *q* = 0.05). (**B**) Aldosterone concentration in blood plasma at steady state (pale colors, bars 1, 3, 5) and after ACTH (synacthen) stimulation (bright colors, bars 2, 4, 6); the data is presented in nM. Green corresponds to the WT (bars 1, 2, *n* = 12, 3 months, male), gray corresponds to *FLAD1*^A418V/A418V^ (bars 3, 4, *n* = 12, 3 months, male), while red corresponds to the *FLAD1*^A418V/R542*^ (bars 5, 6, *n* = 12, 3 months, male) mice. Statistical analysis was performed using a mixed-effects model (REML): significant effects of ACTH (synacthen) stimulation [F(1,15) = 236.0, *P* < 0.0001], Genotype [F(2,33) = 48.21, *P* < 0.0001], and ACTH×Genotype interaction [F(2,15) = 73.90, *P* < 0.0001]. Post hoc: Benjamini, Krieger, and Yekutieli procedure (5 families, *q* = 0.05). (**C**) *POMC* mRNA quantitation by qPCR in the pituitary gland extracts, normalized by mRNA *GAPDH*. Log scale, arbitrary units. *P* = 0.034, Kruskal-Wallis statistic = 6.671 by Kruskal-Wallis test with the Benjamini-Krieger-Yekutieli correction for multiple comparisons. (**D**) H&E staining of adrenal gland samples from the WT (left panel, 4 months, male), *FLAD1*^A418V/A418V^ (central panel, 4 months, male), and *FLAD1*^A418V/R542*^ (right panel, 4 months, male) mice. Scale bar: 1mm. The cortex and medulla zones are marked. (**E**) Time course of the blood glucose concentration (mM) in mice following i.p. injection of 0.75 mU/g mouse weight after 4 hours of fasting. Time points 0, 15, 30, 45, 60, and 120 minutes are indicated below the graphs. Mice genotypes are shown above the graphs: WT (left part, *n* = 6, male, 3 months), *FLAD1*^A418V/A418V^ (central part, *n* = 6, male, 3 months), and *FLAD1*^A418V/R542*^** (right part, *n* = 6, male, 3 months) mice. Data are shown as mean ± 95% CI. Multiple comparisons were corrected using the 2-stage linear step-up procedure of Benjamini, Krieger, and Yekutieli (*q* = 0.05).

To investigate cortical steroidogenesis in the mouse lines carrying FLAD1^A418V/R542*^ and FLAD1^A418V/A418V^ variants, we determined concentrations of adrenal steroids in blood plasma at steady state (Figure 5, A and B, and Supplemental Figure 4, A–C) and after ACTH (synacthen) stimulation (Figure 5, A and B, and Supplemental Figure 5, A–C). We observed that FLAD1^A418V/R542*^ mutations in mice decrease steady-state aldosterone level (Figure 5B) and corticosterone (Figure 5A), but not that of progesterone (Supplemental Figure 5A), 11-deoxycorticosterone (Supplemental Figure 5B), or 18-hydroxycorticosterone (Supplemental Figure 5C). FLAD1^A418V/R542*^ mutant mice demonstrated a decreased or even undetectable response of corticosterone, aldosterone, and 18-hydroxycorticosterone levels for ACTH stimulation (Figure 5, A and B, and Supplemental Figure 5C). For FLAD1^A418V/A418V^ mutant, which has a much milder phenotype, we observed somewhat reduced response to ACTH only for aldosterone (Figure 5B).

Corticosteroids regulate ACTH production via a negative-feedback mechanism that acts through transcriptional repression (29). To check whether adrenal insufficiency caused by *FLAD1* variants may lead to transcriptional depression of the POMC gene, which encodes the ACTH precursor, we extracted total RNA from the pituitary glands of mice and analyzed gene expression by qPCR (Figure 5C). As expected, we observed an upregulation of the POMC gene in mice carrying FLAD1^A418V/R542*^ and FLAD1^A418V/A418V^ genotypes.

To determine whether FAD deficiency affects adrenal gland morphology, we performed H&E staining (Figure 5D). *FLAD1*^A418V/A418V^ mice’s adrenal gland morphology appeared normal. However, mice of the *FLAD1*^A418V/R542*^ genotype appeared to have reduced and disorganized the entire cortical region (Figure 5D).

### The variants in the FLAD1 gene cause age-dependent hypoglycemia.

The patients described herein had low-normal blood glucose levels. Patients with FLAD1 variants described in the literature were occasionally reported to have hypoglycemia(16, 20, 25). To compare it with the mouse model, we performed an insulin tolerance test (ITT) in WT mice and mice carrying the FLAD1^A418V/R542*^ and FLAD1^A418V/A418V^ genotypes (Figure 5E and Supplemental Figure 5). At 3 months of age, mice responded to insulin injection in a manner highly dependent on genotype (Figure 5). WT mice and FLAD1^A418V/A418V^ mutants reacted normally, while mice carrying the FLAD1^A418V/R542*^ genotype demonstrated a substantial delay in glucose level normalization and a more severe glucose concentration drop. Later, at 6 months of age, FLAD1^A418V/R542*^ mutants were found to have a smaller difference with the WT and FLAD1^A418V/A418V^ mutants in the insulin-provoked glucose concentration drop, while this difference remained statistically significant (Supplemental Figure 6A). Insulin-provoked corticosterone release (Supplemental Figure 6B) was barely, if at all, observed for FLAD1^A418V/R542*^ mutants. In line with other observations, FLAD1^A418V/^ ^A418V^ mutants behaved much like the WT mice in this test.

## Discussion

Patients with *FLAD1* variants differ in symptom onset time and severity, even with the same variants (Supplemental Table 2). Major symptoms include hypotonia, exercise intolerance, swallowing and sucking difficulties (22), and sometimes scoliosis (13, 15). If lethal, the deficiency of FLAD1 leads to lethality at several months of age, mainly due to respiratory disease (13, 24). At the same time, some patients demonstrated symptom onset only in adulthood and remained alive beyond age 50 years (13, 19). The standard treatment for FLAD1 insufficiency includes high doses of riboflavin, which is usually helpful, especially in less severe cases (13, 17, 19, 25).

Clinical manifestations of the *FLAD1* variants described herein are largely similar to those of previously reported cases (Supplemental Table 2). On the biochemical level, the most prominent is an increased concentration of fatty acids and acyl carnitines in the blood. Both patients described experiencing severe pneumonia at an early age. Pulmonary insufficiency is a primary cause of mortality in patients carrying *FLAD1* variants (13, 24); fortunately, it was not the case for our patients. Similar to the other published cases with childhood onset, the patients presented with muscle hypotonia, which most likely was the cause of the respiratory disease during infancy and later led to the development of scoliosis. In the literature, we have not encountered any allusions to hypocortisolism in the context of FLAD1 deficiency; we could not find any reports in which adrenal function was evaluated and found to be normal. We do not have access to other patients with FLAD1 mutations, as this deficiency is rare, with only a handful of cases reported worldwide. In general, in the case of deficiency of redox partner proteins, one might expect variable steroidogenic phenotypes. For instance, only a minority of patients with POR deficiency present with overt adrenal crisis, and its risk seems to be increased in patients with certain pathogenic variants (30). It is likely that epistatic genetic factors contribute to the variability in symptoms. Also, environmental factors, such as the availability of flavins and differences in dietary supply of enzymatic substrates whose metabolism depends on FAD, may affect clinical manifestations. Hypothetically, an additional mutation-specific FAD transfer defect from FLAD1 to steroidogenic AdR might be considered. Finally, since we observed a reduction and disorganization of the entire cortical area of adrenal glands in FLAD1^A418V/R542*^ mutant mice, a defect in the development of this zone caused by FAD limitation might be the primary cause of adrenal insufficiency, rather than a specific influence on a particular steroidogenic enzyme. Further experiments might be needed to mechanistically understand the differences in adrenal phenotypes of FLAD1 mutations.

*FLAD1* mutations affect estrous cycle length in female mice but do not affect males’ ability to impregnate females. Thus, adrenal steroidogenesis is likely to be primarily affected by the mutations, with some influence on gonadal steroidogenesis not excluded. The observed effects on adrenal steroidogenesis could be explained by the following main principles. *FLAD1*^A418V/R542*^ compound heterozygous mutations demonstrate a much more severe phenotype in comparison with the homozygous *FLAD1*^A418V/A418V^ mutation. The higher the demand for FAD in a particular process or condition, such as a boost in steroidogenesis by ACTH stimulation, the greater the difference observed between WT and mutant mice. While all adrenal steroidogenic pathways are affected by the mutation, starting from the cholesterol side-chain cleavage by CYP11A1, the concentrations of compounds requiring additional sequential FAD-dependent hydroxylation steps depend more heavily on FLAD1 function. The steps of mitochondrial steroidogenesis are more affected, consistent with earlier published data on the severe mitochondrial dysfunction caused by FLAD1 insufficiency (31). Interestingly, in patients, cortisol concentration was primarily affected by *FLAD1* mutations, whereas in mice, aldosterone concentration was more affected. It is likely because, in humans, cortisol formation requires an additional hydroxylation step that is not present in mice. Mice’s major stress mediator, corticosterone, requires fewer FAD-dependent steps for its biosynthesis.

The allele frequencies of *FLAD1* pathogenic variants (Supplemental Table 1) range from 2 × 10^–5^ to 7 × 10^–7^, making the development of the corresponding disease in a case of homozygous or compound heterozygous variant genotypes rare. The majority of pathogenic variants in the *FLAD1* gene are frameshift variants in the molybdopterin-binding N-terminal domain (Figure 1A). It is likely that these variants do not inactivate FAD synthase activity completely due to the presence of an hFADS6 isoform devoid of the N-terminal domain.

The sole animal model for FLAD1 deficiency developed so far was *C*. *elegans* with siRNA-silenced FADS. This model demonstrates growth slowing, mitochondrial unfolded protein response (mUPR), likely due to denaturation of apoflavoproteins, and decreased content of flavins. The locomotion defect and flavin content could be reversed by a riboflavin supplement (32). The only mouse model for MADD is a missense *Etfdh* A84T/A84T mutant mouse line (33), which is clinically and biochemically similar to patients with riboflavin-responsive MADD (RR-MADD). Although the *ETF* variant does not recapitulate all features of *FLAD1* variants, since it affects only fatty acid oxidation, it does not affect, for example, steroid hormone biosynthesis, as observed here. In contrast, FLAD1 deficiency affects the entire FAD-containing proteome, encompassing over 90 proteins (34). It is likely that the mouse model has not been developed earlier due to the lethality of homozygous *FLAD1* inactivation.

The personalized mouse model carrying the compound heterozygous *FLAD1* variants A418V and R542* recapitulates the phenotypes observed in patients, as described earlier in the literature (Supplemental Table 2). Among them are excess blood fatty acids, acyl carnitines, and muscle weakness. We also observed a 1.7-fold decrease in FAD concentration in adrenal gland extracts from *FLAD1*^A418V/R542*^ mice, roughly matching that reported in other studies. FAD synthesis rates published for the fibroblasts of patients carrying variants Ser495del, Val191Glnfs*10/Arg530Cys, and Ala176Glnfs*8 fall 2- to 4-fold relative to those of control fibroblasts, while total concentration of FAD remained within the normal range (13). At the same time, a patient with *FLAD1* Arg249* variant resulted in a 10-fold reduction in FAD synthesis rate and ~2-fold reduction in FAD content relative to the control fibroblasts (25).

A personalized mouse model carrying the *FLAD1*^A418V/R542*^ genotype also recapitulated adrenal insufficiency in the patients. We observed a decreased steady-state level of aldocosterone and a near absence of corticosterone and aldosterone release after ACTH injection, together with transcriptional upregulation of the ACTH precursor gene, *POMC*.

Thus, we created the mouse model for FLAD1 deficiency, recapitulating both typical biochemical manifestations and adrenal insufficiency. We proved that previously undescribed variants in the *FLAD1* gene, A418V and R542*, are causative of the disease manifestation in patients. The A418V variant is hypofunctional and tolerable, while R542* is lethal in the homozygous state. The development of a mouse model for the disease paves the way for investigating the molecular mechanisms underlying the pathology and further improving therapeutic interventions.

### Limitations of the study

The mouse model we presented in this manuscript has a few potential limitations. The difference is in steroidogenic pathways in humans and mice. In humans, the main glucocorticoid hormone is cortisol, while in rodents, including mice, due to the lack of 17ɑ-hydroxylase, corticosterone is used instead. This may lead to a difference between the mouse model and patients. Furthermore, only a statistically insignificant decrease in bone density has been observed for the *FLAD1*^A418V/R542*^ mice, while the patients demonstrated severe scoliosis. The latter, however, may reflect different effects of muscle weakness on vertebral function in rodents and upright walking men. The unresolved question is whether adrenal insufficiency is general to other FLAD1 mutants or specific for the *FLAD1*^A418V/R542*^ mutations tested in this work. Mechanistic understanding of the influence of the FLAD1^A418V/R542*^ mutations on steroidogenesis would require further experiments.

## Methods

### Sex as a biological variable.

The clinical study included both male and female siblings. For mouse experiments, male mice were used preferentially, as they exhibit less hormonal variability between individuals compared with female mice, whose estrous cycles can confound data interpretation. Female mice were included exclusively in the estrous cycle study. Given that the findings are expected to be relevant to both sexes, we believe this approach does not limit the generalizability of the results.

Reagents and tools are listed in Supplemental Table 3.

### DNA analysis in the patients.

DNA samples from peripheral leukocytes were extracted using the PureLink Genomic DNA Mini Kit (Thermo Fisher Scientific). Sanger sequencing was performed on the 3500xL Genetic Analyzer (Thermo Fisher Scientific).

### Whole exome sequencing (Patient 1).

Library preparation was done with the Ion AmpliSeq Exome RDY Kit (Thermo Fisher Scientific) according to the manufacturer’s protocol. Ion library TaqMan quantitation kit (Thermo Fisher Scientific) was used for library quantitation. The sequencing was performed on the Ion Proton Sequencer (Thermo Fisher Scientific). Data analysis was carried out using Ion Torrent Suite 1 version 5.4 (Thermo Fisher Scientific) and ANNOVAR ver. 2018Apr16 software packages.

For amplicon sequencing, a 3,167 bp genomic DNA fragment (hg19_chr1:154962123_154965289) was amplified (see Supplemental Table S3 for primer sequences) and cloned into the pGEM-T Easy vector (Promega). Individual clones were subjected to Sanger sequencing.

### Mice.

Experiments with mice were conducted in strict compliance with national and international guidelines for the Care and Use of Laboratory Animals. The animal study was carried out in accordance with the ARRIVE guidelines. The work with animals was approved by the animal ethics committee of the A.N. Belozersky Institute of Physico-Chemical Biology of the Lomonosov Moscow State University, protocol no. 018-3/07/2025.

Animals were kept in individually ventilated cages (IVC system, TECNIPLAST S.p.A.) with free access to food and water purified by reverse osmosis, in an environment free of specific pathogens, with a light regime 12/12 (light on at 09:00), in rooms with an air exchange rate of more than 15 r/h, at 20°C–24°C, humidity 30%–70%. Wood chips with minimal dust formation were used as bedding. Shelters and nest-building materials made of natural materials were used as environmental enrichment. All materials supplied to the animals were sterilized by autoclaving.

All invasive procedures were carried out under anesthesia, either isoflurane inhalation (35) or general anesthesia using a Zoletil-Xylazine combination (36). The animals were euthanized under general anesthesia with subsequent cervical dislocation according to NIH guidelines (37).

### Mice genome editing.

Desired mutations were introduced by the CRISPR/Cas9 system. sgRNA was designed using https://chopchop.cbu.uib.no web resource (38). sgRNA was obtained by in vitro T7 transcription (Thermo Fisher Scientific) from PCR-amplified templates generated from the plasmid pX458 (39) (see key resource Table for primer sequences). The obtained sgRNA was mixed with Cas9 mRNA (Thermo Fisher Scientific) and diluted in the filtered microinjection buffer (10 mM Tris, 0.1 mM EDTA, pH 8) to final concentrations of 25 ng/μL sgRNA, 50 ng/μL Cas9 mRNA, and 10 ng/μL ssDNA template for homologous recombination (see Supplemental Table S3 for primer sequences).

C57BL/6J and CBA mice (Federal Research Center Institute of Cytology and Genetics, Siberian Branch, Russian Academy of Sciences [ICG SB RAS], Novosibirsk, Russia), with minimal disease status, were mated to obtain F1 hybrids (C57BL/6J × CBA). Zygotes were obtained by mating the hybrid superovulated female mice with males using a standard procedure (40). As many as 961 zygotes were microinjected into the cytoplasm and transferred into the oviducts of pseudo-pregnant female F1 hybrids C57BL/6J × CBA mice. Out of 16 pups born in the experiment aiming at the A418V mutation, 1 contained the desired mutation. In a similar experiment targeting the R542* mutation, 3 pups were born; 1 carried the desired mutation. To avoid potential confounding effects of unlikely secondary mutations, the founder pups were backcrossed to the C57BL/6J line. Mice C57BL/6J *FLAD1*^A418V/A418V^ and C57BL/6J *FLAD1*^A418V/R542*^ were obtained by breeding of C57BL/6J *FLAD1*^WT/A418V^, C57BL/6J *FLAD1*^A418V/A418V^, and C57BL/6J *FLAD1*^WT/R542*^ mice.

### Genotyping.

Genotyping of the mice was performed using tiny ear pieces, which were extracted with QuickExtract DNA Extraction Solution (Lucigen) according to the manufacturer’s instructions, and analyzed by PCR with Taq DNA polymerase (Evrogen) (see Supplemental Table S3 for primer sequences) and by Sanger sequencing of the amplicon.

Immunoblotting. Immunoblotting of tissue lysates was performed as previously described (41). For immunoblot analysis, samples of adrenal gland, red, and white muscle tissue were lysed in lysis buffer (5% SDS, 0.1% SDC, 100 mM TEAB) supplied with protease inhibitor cocktail (Thermo Fisher Scientific). Obtained cell lysates were applied to the 12% SDS-PAAG, followed by wet transfer to the PVDF membrane (0.45 μm, Thermo Fisher Scientific). Subsequently, the membrane was blocked for 1 hour in TBST (10 mM Tris-HCl, pH 7.5, 150 mM NaCl, 0.1% Tween-20) containing 5% bovine serum albumin (BSA, Proliant Biologicals). Primary antibodies anti-FLAD1 were diluted 1:2000 in TBST containing 5% BSA. The secondary HRP-conjugate anti-rabbit antibodies were used at a 1:5,000 dilution. Anti-GAPDH (1:10,000) antibodies were used as a loading control.

### qPCR.

For the measurement of POMC level, total RNA was extracted from the whole mouse hypophysis with RNA extract reagent (Evrogen), followed by cDNA synthesis with either a Maxima First Strand cDNA Synthesis Kit for qPCR (Thermo Fisher Scientific) with a random hexamer primer. qPCR was performed using Hot Start Taq DNA polymerase (Evrogen) in the presence of SYBR Green. The following primers were used to amplify POMC mRNA (Supplemental Table 3).

### FAD/FMN/Riboflavin content.

Individual mouse adrenal glands were homogenized in 100 μL of distilled water with a Potter homogenizer. Unbroken tissues and cell debris were removed by centrifugation at 400*g* (10 minutes). Proteins in the obtained supernatant were precipitated with 5% (v/v) trifluoroacetic acid and sedimented by centrifugation. The supernatant was neutralized with K_2_HPO_4_ and used to determine FAD, FMN, and riboflavin concentrations. Flavins were separated by reverse-phase HPLC using a Zorbax SB-С18 column (4.6 × 150 mm). A linear gradient of acetonitrile (ranging from 10% to 70% v/v, 25 min) in 5 mM MES-Tris (pH 6.0) with a flow rate of 0.2 mL/min was used. Flavins were detected by fluorescence with excitation at 445 nm and emission at 525 nm. Concentrations of flavins were determined using calibration with FAD, FMN, and riboflavin standards of known concentrations. Retention times for FAD, FMN, and riboflavin were 9.7, 13.1, and 18.1 minutes, respectively. Protein concentration was determined by a bicinchoninic acid (BCA) method using bovine serum albumin as a standard.

### CT imaging and evaluation of vertebral curvature and bone density in mice.

High-resolution 3D scans of the mouse spine were obtained using the IVIS Spectrum CT system (PerkinElmer) (42). CT imaging was performed using a standard mouse CT sequence. During CT imaging, mice were anesthetized with an isoflurane/air (2%/98%) mixture. Quantitative analysis of CT datasets was performed with Living Image v. 4.4 (PerkinElmer). Spinal curvature was assessed relative to the T8, T10, T12, and L5 vertebrae, while bone density was quantified through Hounsfield unit measurements (43).

### Assessment of grip strength.

The test is designed to evaluate maximum voluntary strength (grip force) and is commonly used to assess limb strength and neurological deficits in mice and rats (44). The experiment was performed after 6 hours of food deprivation. The mouse is gently pulled back by its tail, ensuring it grips the top portion of the grid, keeping its torso horizontal, and recording the maximum grip strength displayed on the screen. This procedure is repeated with 10-second intervals between trials until 10 results are obtained. After selecting the 5 highest values, the mean is calculated.

### The ITT.

Insulin sensitivity was evaluated according to the published protocol (45). For the ITT, 0.75 mU/g human insulin was administered by i.p. injection to mice that had been fasted for 6 hours, and blood glucose levels were measured over the next 2 hours. Blood glucose levels were measured at 15, 30, 45, 60, and 120 minutes after injections using Test Strips of a point-of-care Accutrend Plus System machine (Roche Diagnostic Australia Pty Ltd.).

### Analysis of whole blood acyl carnitine profile.

Blood was collected via submandibular vein puncture in anesthetized mice, with samples fixed on polystyrene plates (46). Blood analysis was performed using tandem mass spectrometry (MS/MS) in accordance with standard operating procedures. A plate template was prepared for the identification of dried blood spots (DBS) samples, blanks, and controls. Using a manual or automated puncher, 3 mm DBS spots, blanks, and controls were punched into the wells of a polystyrene plate according to the predefined template. The daily solution was prepared from internal standards as follows: 50 μL each of the amino acid standard and the acyl carnitine standard (stored at –20°C throughout the entire expiration date indicated on the package) were added to 9.9 mL of extraction solution, followed by gentle mixing. Using a multichannel pipette, 90 μL of the daily solution was dispensed into each well of the plate containing DBS samples. The plate was sealed with an adhesive film and incubated for 30 minutes at 30°C with orbital shaking (650–750 rpm). After incubation, the adhesive film was removed, and 75 μL of each sample was transferred to a new polypropylene plate. Samples were dried under a stream of nitrogen or air, and then 50 μL of 3N HCl in butanol was added. The plate was resealed using heat-stable film with a plate sealer and incubated for 30 minutes at 60°C. After incubation, the film was removed, and samples were dried again under nitrogen or an air stream. Next, 75 μL of Reconstruction Solution (Perkin Elmer Life and Analytical Sciences) was added to each well, and the plate was sealed with aluminum foil. The plate was incubated for an additional 10 minutes at 27°C (650–750 rpm). Following incubation, the plate was loaded into the autosampler. Before analysis, the mobile phase (flow solvent) was verified to ensure sufficient volume for the run, after which the analysis was initiated via Analyst software.

### The estrous cycle stages.

Estrous cycles of female mice from all 3 lines (7 mice per group) were determined by analyzing the cellular composition of vaginal smears, collected daily at the same time of day for 2 weeks. The cycle is divided into 4 stages: proestrus (less than 24 hours), estrus (12–48 hours), metestrus (8–24 hours), and diestrus (48–72 hours), with an average total cycle length of 4–5 days (47). The following parameters were assessed: number of diestrus days (diestrus prolongation may indicate cycle disruption and reduced fertility), estrous cycle length (frequency of estrus occurrence), and cycle regularity.

### Male fertility assessment.

To evaluate male mice fertility, *FLAD1*^A418V^ (*n* = 8), *FLAD1*^A418V/R542*^ (*n* = 8), and WT male mice (*n* = 8) were housed individually for 10 consecutive nights. Each evening at 18:00, a single male mouse was placed in a cage with 2 WT female mice that had never been exposed to males. The following morning (11:00–12:00), female mice were removed and examined for vaginal plugs. Any female mouse with a vaginal plug was excluded from further testing and replaced with a naive WT female mouse. Each day, male mice were paired with a new, randomized pair of WT female mice. After 10 nights, the resulting data were collected and analyzed.

### Functional assessment of the adrenal cortex.

Assessment of adrenal insufficiency was performed via evaluation of ACTH-stimulated corticosterone concentrations in mouse blood. The lack of a corticosterone response to Synacthen administration (a synthetic ACTH analog containing the first 24 amino acids of ACTH while retaining the full biological potency of the endogenous peptide) indicates adrenal cortex dysfunction. Quantitative analysis of serum aldosterone, progesterone, 11-deoxycorticosterone, corticosterone, and 18-hydroxycorticosterone levels was performed by LC-MS/MS as previously described, at baseline and following stress induction with 100 μg/kg Synacthen (48, 49).

The sample preparation method was based on the previously described procedure with some modifications (49). In total, 20 μL of blood plasma were transferred to Eppendorf plastic microtubes. Ten microliters of precipitant, containing a 1:1 mixture of internal standards in methanol and 1.2 microliters of zinc sulfate at a concentration of 1.25M, were added. The samples were shaken at 2,300 rpm for 5 minutes at room temperature, then centrifuged at 20,300*g* for 5 minutes at 10°C. Forty microliters were then transferred to a 96-well microplate. Samples were analyzed on the day of preparation.

The sample preparation method was based on the previously described procedure with some modifications (49). Twenty microliters of blood plasma were transferred to Eppendorf plastic microtubes. Ten microliters of precipitant, containing a 1:1 mixture of internal standards in methanol and 1.2 μL of zinc sulfate at a concentration of 1.25M, were added. The samples were shaken at 2,300 rpm for 5 minutes at room temperature, and then centrifuged at 20,300*g* for 5 minutes at 10°C. Forty microliters were then transferred to a 96-well microplate. Samples were analyzed on the day of preparation.

The I-Class liquid chromatography system (Waters) consisted of a binary pump (BSM) and a quaternary pump (QSM), an autosampler (FTN-I), and a column thermostat (CM-I) equipped with a 2-position, 6-port valve for the trap-and-elute method. A Xevo TQ-XS tandem triple-quadrupole mass spectrometer (Waters) with an APCI ionization source operating in dual-polarity multiple reaction monitoring (MRM) mode was used as the detector. Source parameters are listed in Supplemental Table 4. Waters ACQUITY UPLC BEH C18 (2.1 x 30 mm, 1,7 μm) was used as a trap column, and Waters XSelect HSS C18 column (3,0 x 75 mm, 2.5 μm) at 40°C was used for the separation of the analytes. Gradient programs can be found in Supplemental Tables 5 and 6. The trap-and-elute method was based on a 6-port 2-position valve in the thermostat (Supplemental Figure 5D). Position 1 changed to position 2 during analysis according to the program provided in Supplemental Table 7. All MRM parameters were defined individually for each analyte and listed in Supplemental Table 8; analyte concentrations in calibrators and quality control samples are provided in Supplemental Table 9. A typical chromatogram of the analyte mixture is shown in Supplemental Figure 5E. Prepared samples were stored in an autosampler at 12°C. Injection volume was set to 20 μL. Water/isopropanol 50:50 mixture was used as a strong sampler needle wash, and water: methanol 50:50 as a weak needle wash.

An analysis of homozygous mouse lines’ embryonic lethality. At all stages of the study, heterozygous mice carrying either FLAD1^wt/A418V^ or FLAD1^wt/R542*^ mutations have been crossed with each other. The initial evaluation focused on the distribution of genotypes across litters. At the next stage, after breeding and confirming mating via vaginal plugs (50), pregnant female mice were individually housed. Embryos were surgically harvested at E0.5, E4.5, E8.5, E11.5, and E14.5 (51, 52). Fertilized zygotes were cultured at 37°C under a 5% CO_2_ atmosphere until reaching the blastocyst stage. Throughout all steps, the genotypes of mice and embryos were analyzed by PCR and Sanger sequencing of the amplicon (53).

### Resource availability.

The mouse model described is available upon request to the Lomonosov Moscow State University biocollection of genome-edited mice.

### Materials availability.

All data is included in the text of the publication and the Supplemental files.

Spelling was corrected by Grammarly (54).

### Statistics.

For all panels, IQRs are shown as solid bars, while the full data ranges are shown as thin lines. The horizontal line represents the median, and the cross represents the mean. Normality of the data was assessed using the Shapiro-Wilk test. Comparisons of a single parameter across genotypes were performed using the Kruskal-Wallis test with the Benjamini-Krieger-Yekutieli correction. For Figure 5, A and B, a 2-way repeated measures (RM) ANOVA was applied, with genotype and time relative to ACTH administration (before and after) as the 2 factors. For the ITT (Figure 5E), a mixed-effects model (REML) was used to account for a missing data point due to the death of 1 animal during the test, as well as for the 2-factor structure of the analysis (genotype and time after insulin injection). For the female fertility experiment (Figure 4C), the probability of vaginal plug detection was compared using the Mantel-Cox test. Detailed sample size information is provided in the respective figure captions or visualized as individual points in the graphs.

All statistical analyses were performed using GraphPad Prism 10.4.0 (Dotmatics) and Microsoft Office Excel (Microsoft Corporation).

Study approval. The animals were euthanized under general anesthesia with subsequent cervical dislocation according to NIH guidelines (37). The work with animals was approved by the animal ethic committee of the A.N. Belozersky Institute of Physico-Chemical Biology of the Lomonosov Moscow State University, protocol No. 018-3/07/2025.

### Data availability.

All data underlying the findings of this study are available within the manuscript and its supplemental materials. Individual-level data supporting the figures are provided in the Supporting Data Values file.

## Author contributions

Conceptualization was contributed by ANT and PVS. Methodology was contributed by OAA, VAI, AV Pirogov, AVB, EAG, OOG, GVB, MAA, and VNM. Investigation was contributed by OAA, NYK, VAI, AV Priymak, AVB, OAP, VSB, EAG, AV Pirogov, OAB, MAE, OOG, MAA, and VNM. Writing of the original draft was contributed by PVS. Review and editing were contributed by PVS, OAA, AVB, OAD, and ANT. Funding acquisition was contributed by PVS. Resources were contributed by OAA, VAI, AV Pirogov, GVB, MAA, VNM, and OAD. Supervision was contributed by OAA, NYK, ANT, and PVS.

## Conflict of interest

The authors declare that they have no known competing financial interests or personal relationships that could have appeared to influence the work reported in this paper.

## Funding support

- Ministry of Science and Higher Education of Russian Federation (agreement no. 075-15-2025-489).

## Supplementary Material

Supplemental data

Unedited blot and gel images

Supporting data values

## Figures and Tables

**Table 1:**
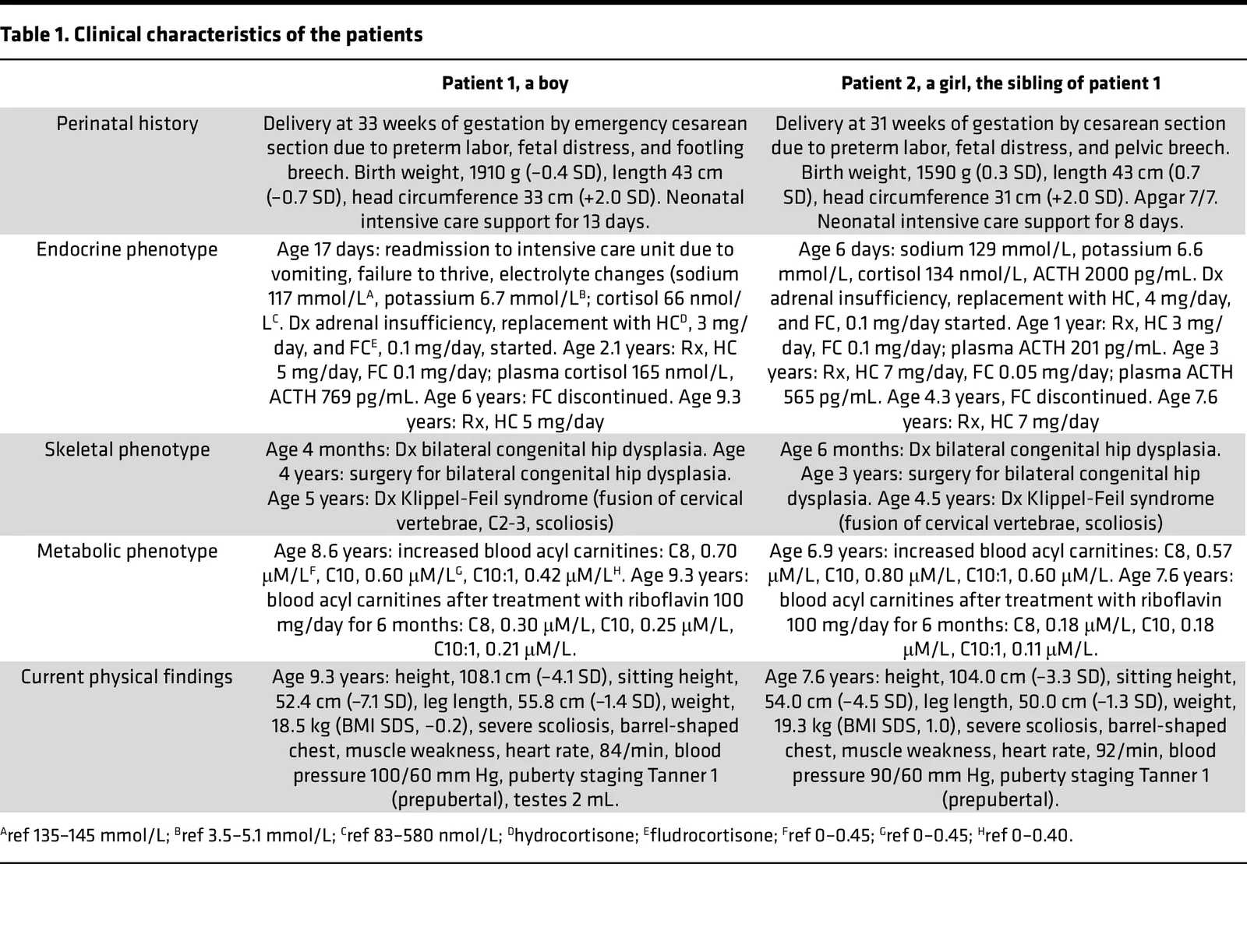
Clinical characteristics of the patients

## Version 1 09/08/2026

Electronic publication
