# Supplementary material for: A familial case of FLAD1 protein deficiency associated with impaired adrenal steroidogenesis
Source: JCI Insight. 2026 Sep 8;11(17):e198799. doi: 10.1172/jci.insight.198799 (PMC13564092; doi:10.1172/jci.insight.198799)
Supplement: Supplementary file 1 [file jciinsight-11-198799-s203.pdf]

SUPPLEMENTARY INFORMATION

A familial case of FLAD1 protein deficiency associated with impaired adrenal steroidogenesis.

Olga A. Averina[1], Natalia Yu. Kalinchenko[2], Vitaly A. Ioutsi[2], Andrey V. Pirogov[3], Alexander V. Bogachev[1], Oleg A. Permyakov[1], Vitaly S. Buev[1,4], Ekaterina A. Guseva[1,3,4], Anastasia V. Priymak[1,3], Olga A. Bazhanova[5], Mariia A. Emelianova[4], Olga O. Grigorieva[1], Galina V. Baydakova[5], Maxim A. Abakumov[6], Vasily N. Manskikh[1], Olga A. Dontsova[1,3,4,7], Petr V. Sergiev[1,3,4], Anatoly N. Tiulpakov[5]

[1]Belozersky Institute of Physico-Chemical Biology, Lomonosov Moscow State University, 119991 Moscow, Russia

[2]Endocrinology Research Centre, 117292 Moscow, Russia.

[3]Faculty of Chemistry, Lomonosov Moscow State University, 119991 Moscow, Russia

[4]Center for Bio- and Medical Technologies, Skolkovo Institute of Science and Technology, 143025 Skolkovo, Russia

[5]Research Centre for Medical Genetics, Moscow 115522, Russia

[6]Pirogov Russian National Research Medical University, 117513 Moscow, Russia

[7]Shemyakin–Ovchinnikov Institute of Bioorganic Chemistry, 117997 Moscow, Russia

Authorship note: OAA and NYuK contributed equally to this work; PVS and ANT contributed equally to this work.

Supplementary Tables

Table S1. *FLAD1* alleles with pathogenic outcomes.

	Citation	Frequency(1)	Predicted pathogenicity	
			PolyPhen2(2)	FATHMM(3)
Arg109Alafs*3	Tylor et al. (4)	—		
Phe134Cysfs*8	Olsen et al. (5); Yildiz et al.(6);	—		
Arg148*	Muru et al.(7)	$4\cdot10^{-6}$		
Ser167Profs*20+ Phe170Leu	Olsen et al. (5)	$7\cdot10^{-7}$		
Ala176Glnfs*8	Olsen et al. (5)	—		
Asp185Val	Hosseini et al.(8)	—	Probably damaging 1	Damaging -13.45
Val191Glnfs*10	Olsen et al.(5); Auranen et al.(9)	$4\cdot10^{-6}$		
Arg249*	Ryder et al.(10); Lee et al.(11); Yamada et al.(12); Tolomeo et al. (13)	$1.7\cdot10^{-5}$		
Glu266Valfs*3	García-Villoria et al.(14)	$1.4\cdot10^{-6}$		
Phe279Serfs*45	Olsen et al. (5)	—		
Ser495del	Olsen et al. (5)	—		
Arg530Cys	Olsen et al.(5); Muru et al.(7); Auranen et al.(9); Vengalil et al.(15); Wen et al.(16)	$2.2\cdot10^{-5}$	Probably damaging 1	Damaging -6.26
Arg530Pro	Wen et al.(16)	$6.6\cdot10^{-6}$	Probably damaging 1	Damaging -4.62
Ala418Val	This study	$5\cdot10^{-6}$	Possibly damaging 0.711	Tolerated -1.25
Arg542*	This study	$1.3\cdot10^{-6}$		

Table S2. Patients with *FLAD1* mutations.

FLAD1 genotype	Age of death (or age alive)	symptoms	Treatment, results	Citation
Homozygous Phe134Cysfs*8	8mo	Lipid myopathy		Olsen et al. (5)
Homozygous Ser495del	3d	Cardiorespiratory collapse		Olsen et al. (5)
Homozygous Ser495del	22 y, alive	Cardiomyopathy in the first year of life	Riboflavin, responsive	Olsen et al. (5)
Val191Glnfs*10/ Arg530Cys	44 y, alive	Exercise intolerance		Olsen et al. (5) Auranen et al. (9)
Phe279Serfs*45/ Arg530Cys	56 y, alive	Exercise intolerance		Olsen et al. (5)
Homozygous Ala176Glnfs*8	8 y, alive	Muscle hypotonia		Olsen et al. (5)
Homozygous Ala176Glnfs*8	16 y	Muscle hypotonia, scoliosis, pneumonia		Olsen et al. (5)
Homozygous Phe134Cysfs*8	7 mo	Hypotonia, cardiac arrest		Olsen et al. (5)
Arg109Alafs*3/ Ser167Profs*20+ p. Phe170Leu	9 mo	Hypotonia, respiratory insufficiency, pneumonia		Olsen et al. (5)
Homozygous Phe134Cysfs*8	4 mo	Severe neonatal hypotonia and muscle weakness, respiratory deterioration		Olsen et al. (5)
Homozygous Phe134Cysfs*8	6 mo	Hypotonia, respiratory insufficiency, pneumonia		Yıldız et al.(6)
Homozygous Phe134Cysfs*8	5 mo	Weak, floppy, no head control		Yıldız et al.(6)
Homozygous Arg249*	8 y, alive	Myopathy, exercise intolerance	Riboflavin responsive	Ryder et al.(10), Tolomeo et al.(13)
Glu266Valfs*3/ 1555-3C>G (splicing defect)	7 y, alive	Psychomotor delay, hypotonia, scoliosis	Riboflavin nonresponsive	García-Villoria et al.(14)
Homozygous Asp185Val	5 y, alive	Hypotonia, exercise intolerance	Riboflavin partially responsive	Hosseini et al.(8)
Homozygous Arg249*	2 y, alive	Hypotonia, weakness in facial and bulbar muscles, vomiting, and respiratory insufficiency	Riboflavin partially responsive	Lee et al.(11)
Arg148*/ Arg530Cys	2 y, alive	Asymptomatic, identified by newborn screening	Riboflavin preventive	Muru et al.(7)

Homozygous pArg530Cys	36 y, alive	Myalgia, bulbar symptoms		Vengalil et al.(15)
Arg530Cys/ Arg530Pro	56 y, alive	Hypotonia, weakness	Riboflavin responsive	Wen et al.(16)
Homozygous Arg249*	3 y, alive	Bulbar palsy, respiratory insufficiency	Riboflavin partially responsive	Yamada et al.(12)
Ala418Val/ Arg542*	9 y, alive	Hypotonia, scoliosis, adrenal insufficiency, low glucose levels, hyponatremia and hypokalemia, and pneumonia at an early age (<1 year)	Riboflavin responsive	This study
Ala418Val/ Arg542*	7 y, alive	Hypotonia, scoliosis, adrenal insufficiency, low glucose levels, hyponatremia and hypokalemia, and pneumonia at an early age (<1 year)	Riboflavin responsive	This study

Table S3. Reagents and tools

REAGENT OR RESOURCE	SOURCE	IDENTIFIER
Antibodies		
Rabbit polyclonal anti-FLAD1	Invitrogen	Cat#PA5-89731;<br>RRID AB_2805750
Mouse monoclonal anti-GAPDH	Abcam	Cat#ab8245;<br>RRID AB_2107448
Goat polyclonal anti-rabbit IgG, HRP-conjugate	Biorad	Cat#1706515;<br>RRID AB_615912
Goat polyclonal anti-mouse IgG, HRP-conjugate	Thermo scientific	Cat#62-6520;<br>RRID AB_2533947
Biological samples		
peripheral leukocytes	Patient 1, Patient 2, Mother of patients 1 and 2, healthy donor	

Chemicals, peptides, and recombinant proteins		
Taq DNA polymerase	Evrogen	PK113L
HS Taq DNA polymerase	Evrogen	PK017L
GeneArt™ CRISPR Nuclease mRNA	Thermo Scientific	A29378
pGEM®-T Easy vector	Promega	A3600
Halt™ Protease Inhibitor Cocktail (100X)	Thermo Scientific	78429
Fatty-Acid Free BSA	Proliant Biologicals	68700
Critical commercial assays		
QuickExtract™ DNA Extraction Solution	Lucigen	QE0905T
MEGAscript™ T7 Transcription Kit	Thermo Scientific	AM1334
Ion AmpliSeq™ Exome RDY Kit	Thermo Scientific	A38264
PureLink® Genomic DNA Mini Kit	Thermo Scientific	K182001
Ion library TaqMan™ quantitation kit	Thermo Scientific	4468802
ExtractRNA	Evrogen	BC032
Maxima First Strand cDNA Synthesis Kit for RT-qPCR	Thermo Scientific	K1641
5X qPCRmix-HS SYBR	Evrogen	PK147L
Experimental models: organisms/strains		

C57Bl/6J	Federal Research Center Institute of Cytology and Genetics, Siberian Branch, Russian Academy of Sciences (ICG SB RAS)	
CBA	ICG SB RAS	
C57Bl/6J *FLAD1*$^{A418V/A418V}$	Lomonosov MSU	This work
C57Bl/6J *FLAD1*$^{WT/R542*}$	Lomonosov MSU	This work
C57Bl/6J *FLAD1*$^{A418V/R542*}$	Lomonosov MSU	This work
F1 hybrids C57Bl/6J × CBA	Lomonosov MSU	In-house breeding
Oligonucleotides		
GCTTCAACGGGGGCAAAGAC	n/a	Human FLAD1 fwd
CCCATTCCCTTCCCCTAATAC	n/a	Human FLAD1 rev
TCCATAGATGTGTGGAGCTG	Lumiprobe, Moscow, Russia	POMC fwd
GGAAGTGACCCATGACGTA	Lumiprobe, Moscow, Russia	POMC rev

AGACCATCGAGACTGCCCTGGCTCAGTACCATCTCT CCCAGCTCTGCGTGGGCTTCAATGGGGGCAAGGAC TGCACCGCACTCCTGCACCTCTTCCATGTAGCCGTG CAGAGGTAAGCCTACCCCTGGGAGCTAAGGCCTCA GAAAGACCCACCTGAAACCTCTCCAGCTCCCCACC CCAGAAAGCACAGCGTCAGCGTG	IDT, Coralville, USA	Mouse FLAD1 A418V HDR template
TGTAATACGACTCACTATAGGCTTACCTCTGCACGG CTGCAGTTTTAGAGCTAGAAATAGCAAG	Lumiprobe, Moscow, Russia	Mouse *FLAD1* A418V sgRNA fwd
AAAAGCACCGACTCGGTGCC	Lumiprobe, Moscow, Russia	sgRNA rev
CAGTACCATCTCTCCCAGCTCT	Lumiprobe, Moscow, Russia	Mouse *FLAD1* A418V seq fwd
GCTGATCACCTAGCTACCTTCC	Lumiprobe, Moscow, Russia	Mouse *FLAD1* A418V seq rev
TGTGGCTGAGGACAGGTCTAGTGACTGATTGTCATT TCCCAGGACTGGACCTACAGAAACATCTGGGAGTTT CTGCGGCAGCTGTTTGTCCCATACTGCATCCTATAT GACTGAGGGTAAGGTGGTTGACGGGCTGGGGTGA GAGGATGCTGGGAAAGAGAGCCCACCCATCACCCA CATCACCCACATGCTCCTGTCTC	IDT, Coralville, USA	Mouse *FLAD1* R542* HDR template
TGTAATACGACTCACTATAGGACTGCATCCTATATG ACAGAGTTTTAGAGCTAGAAATAGCAAG		Mouse *FLAD1* R542* sgRNA fwd
CCAGGACTGGACCTACAGAAAC		Mouse *FLAD1* R542* seq fwd

AGGGATGTGTACCTGGAGTGTT		Mouse *FLAD1* R542* seq rev
Software and algorithms		
Web tools for selecting target sites for CRISPR/Cas9	chopchop.cbu.uib.no	version 3
Ion Torrent Suite 1	Thermo Scientific	version 5.4
ANNOVAR	https://annovar.openbioinformatics.org	ver. 2018Apr16
Living Image software	PerkinElmer, USA	version 4.4.
Other		
3500xL Genetic Analyzer	Thermo Scientific	
PVDF Transfer Membranes, 0.45 μm	Thermo Scientific	88518

Table S4. Ion source parameters for steroid hormones mass spectrometry

Parameter	Value
Corona voltage	2,3 kV
Cone voltage	40 V
Desolvation gas flow	800 L/hr
Cone gas flow	200 L/hr
Nebuliser pressure	3.0 Bar
Collision Gas Flow	0,11 mL/min
APCI probe temperature	550 °C

Table S5. Quaternary pump gradient program

Flow rate: 0.4 mL/min

Channel A: water, Channel B: methanol

Time, min	A, %	B, %
0	75	25
0,85	75	25
0,90	5	95
10,80	5	95
11,00	5	95
14,00	5	95
14,20	75	25
15,00	75	25

Table S6. Binary pump gradient program

Flow rate: 0.4 mL/min

Channel A: methanol:water=50:50, Channel B: acetonitrile

Time, min	A, %	B, %
0	100	0
0,7	100	0
7,0	90	10
13,5	40	60
13,8	10	90
15,2	10	90
15,4	100	0
16,0	100	0

Table S7. 2-position 6-port valve program

**Time, min**	**Position**
0	1
1.0	2
13.2	1

Table S8. MRM parameters

No	Analyte	Q1 mass	Q3 mass	Collision energy, eV	Cone voltage, V
1	Corticosterone	347.2	121.0	24	26
		347.2	91.0	35	26
2	18-OH-Corticosterone	363.2	269.2	20	22
		363.2	121.0	25	22
3	11-Deoxycorticosterone	331.2	97.0	24	20
		331.2	109.0	30	20
4	Progesterone	315.2	109.1	24	40
		315.2	97.1	22	40
5	Aldosterone (negative ions)	359.2	189.1	17	40
		359.2	331.1	15	40
6	Corticosterone-d_8 (IS)	355.2	337.2	13	26
7	Progesterone-$^{13}C_3$ (IS)	318.2	100.1	22	40
8	Testosterone-d_3 (IS)*	292.2	97.1	26	42
9	18-OH-Corticosterone-d_4 (IS)	367.2	273.2	17	40
10	Aldosterone-d_4 (negative ions)	363.2	335.1	15	40

** Testosterone-d_3 was used as an internal standard for 11-Deoxycorticosterone*

Table S9. Analyte concentrations in calibrators and quality control samples (nmol/L)

	Aldosterone	18-OH-Corticosterone	Corticosterone	11-Deoxycortico-sterone	Progesterone
C1	0.70	2.16	4.51	0.89	1.12
C2	1.41	4.31	9.02	1.77	2.24
C3	2.82	8.62	18.04	3.55	4.47
C4	5.64	17.24	36.08	7.09	8.94
C5	11.27	34.49	72.16	14.18	17.89
C6	22.54	68.97	144.31	28.37	35.77
C7	45.08	137.95	288.63	56.74	71.55
C8	90.17	275.89	577.25	113.48	143.10
C9	180.33	551.79	1154.50	226.96	286.20
C10	360.66	1103.57	2309.00	453.91	572.39
LQC	2.11	6.47	13.53	3.33	3.35
MQC	90.17	275.89	577.25	142.19	143.10
HQC	135.25	413.84	865.88	213.28	214.65

Supplementary figures

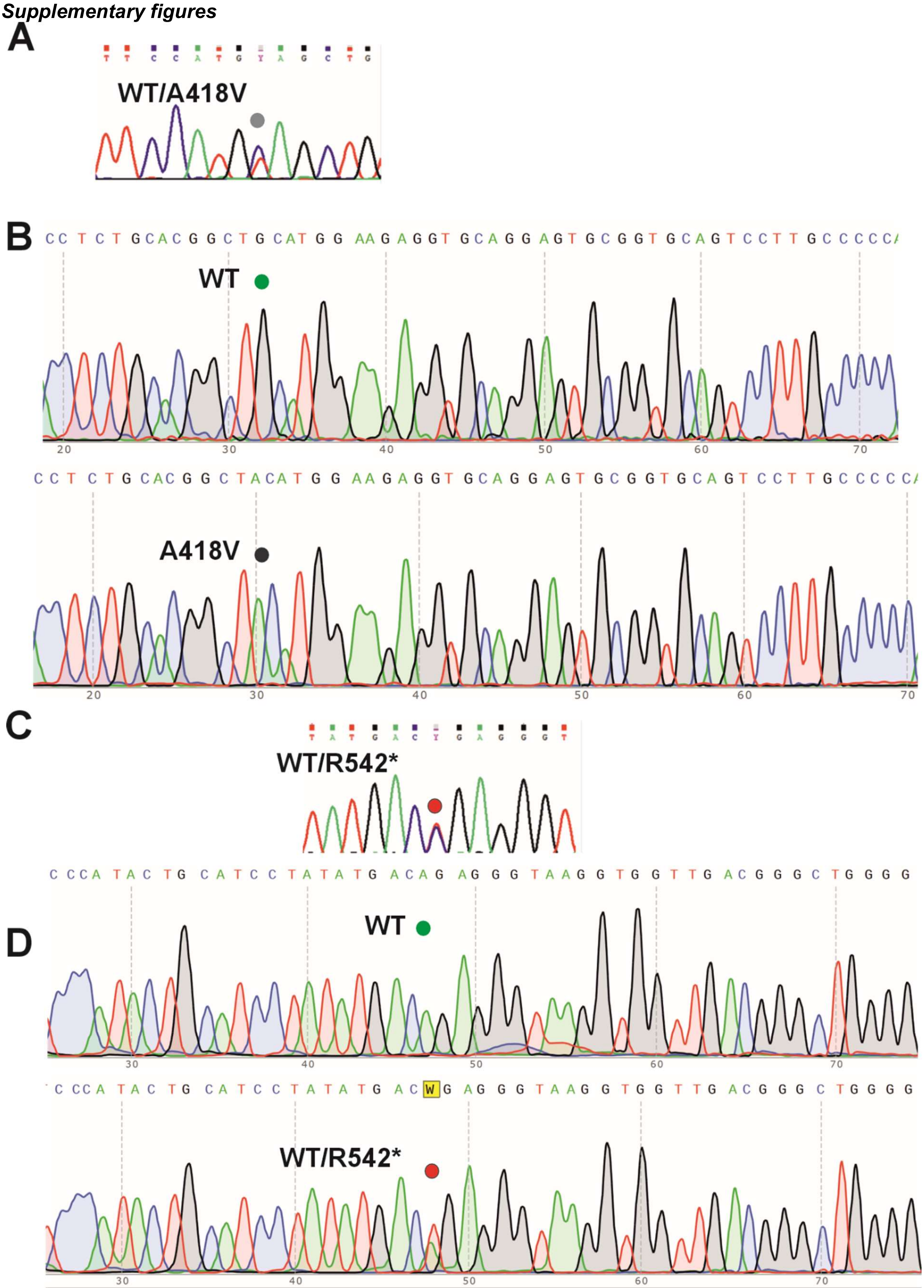

Figure S1. The sequence of the *FLAD1* allelic variants created. (**A**) Sequence of the human *FLAD1* gene in the region coding for amino acid 418. The upper panel corresponds to the wild-type allele, the lower panel to the patient. (**B**) Sequence of the murine *FLAD1* gene in the region coding for amino acid 418. The upper panel corresponds to the wild-type allele, the lower panel to the patient. (**C**) Sequence of the human *FLAD1* gene in the region coding for amino acid 542. The upper panel corresponds to the wild-type allele, the lower panel to the patient. (**D**) Sequence of the murine *FLAD1* gene in the region coding for amino acid 542. The upper panel corresponds to the wild-type allele, and the lower panel to the patient.

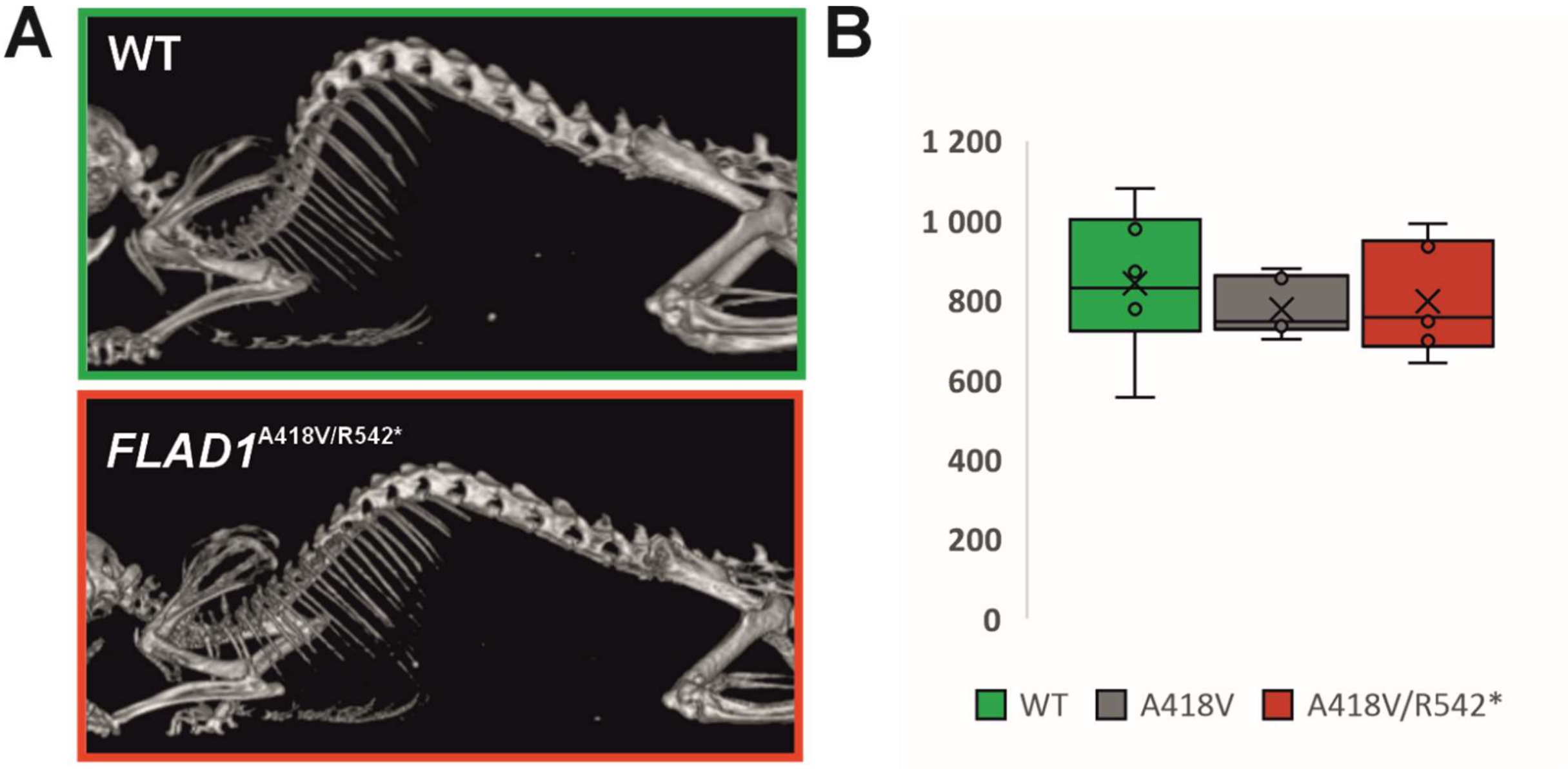

Figure S2. *FLAD1* mutations do not cause scoliosis in mice. (**A**) Tomography of the wild-type mice (upper panel) and *FLAD1*$^{A418V/R542*}$ mice (lower panel) demonstrating the skeleton. (**B**) Bone density by Hounsfield scale. Green color corresponds to the wild type (n = 6, 4Mo, male), gray color corresponds to *FLAD1*$^{A418V/A418V}$ (n = 6, 4Mo, male), while red color corresponds to the *FLAD1*$^{A418V/R542*}$ mice (n = 6, 4Mo, male).

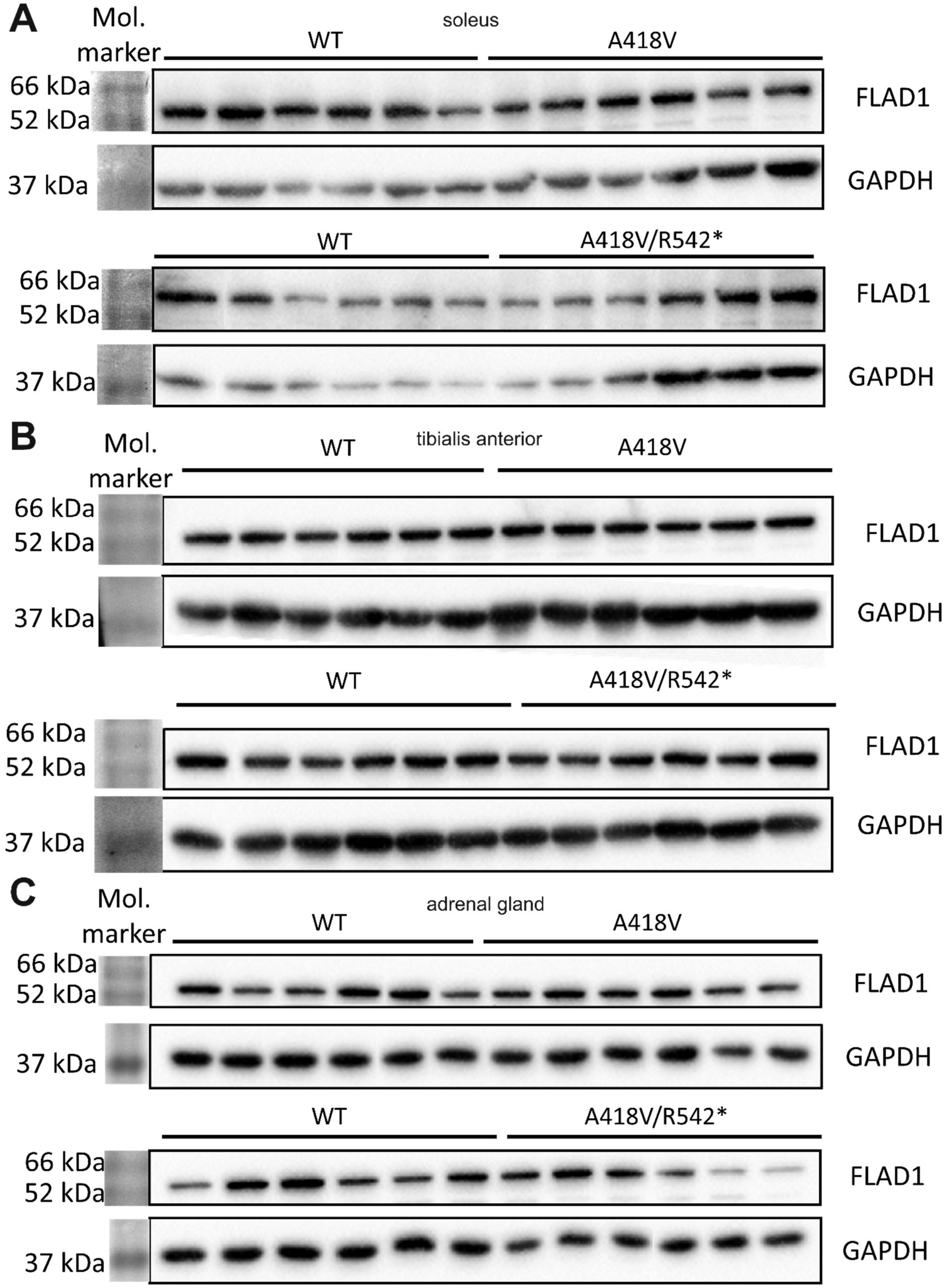
A
Mol.
marker
soleus
WT
A418V
66 kDa
52 kDa
FLAD1
37 kDa
GAPDH
WT
A418V/R542*
66 kDa
52 kDa
FLAD1
37 kDa
GAPDH
B
Mol.
marker
tibialis anterior
WT
A418V
66 kDa
52 kDa
FLAD1
37 kDa
GAPDH
WT
A418V/R542*
66 kDa
52 kDa
FLAD1
37 kDa
GAPDH
C
Mol.
marker
adrenal gland
WT
A418V
66 kDa
52 kDa
FLAD1
37 kDa
GAPDH
WT
A418V/R542*
66 kDa
52 kDa
FLAD1
37 kDa
GAPDH

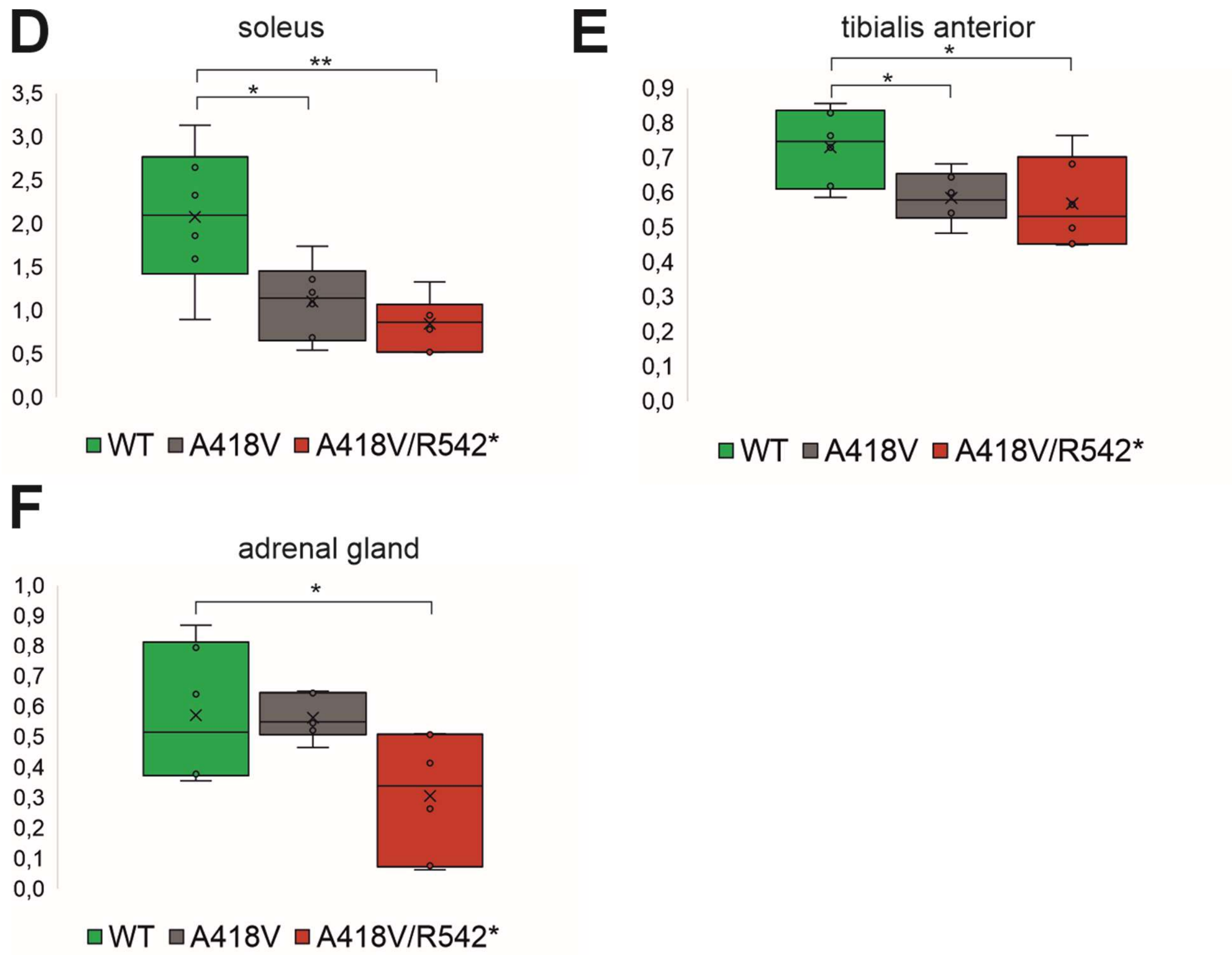

Figure S3. FLAD1 quantity in mouse tissues with mutated FLAD1. Immunoblotting of the soleus (**A**) and tibialis anterior (**B**) muscle extracts and adrenal gland extracts (**C**) with anti-FLAD1 (upper panels) or anti-GAPDH (lower panel, loading control) antibodies. The *FLAD1* genotypes of the mice analyzed are indicated above the lanes as follows: WT (n = 6, 4Mo, male), A418V (n = 6, 4Mo, male), and A418V/R542* (n = 6, 4Mo, male). (**D-F**) Quantification of the immunoblotting results by densitometry. The values provided are normalized by GAPDH. The green color corresponds to the wild type, the gray color to $FLAD1^{A418V/A418V}$, and the red color to $FLAD1^{A418V/R542*}$ mice. *p < 0.05, **p < 0.01 by Mann-Whitney U-test.

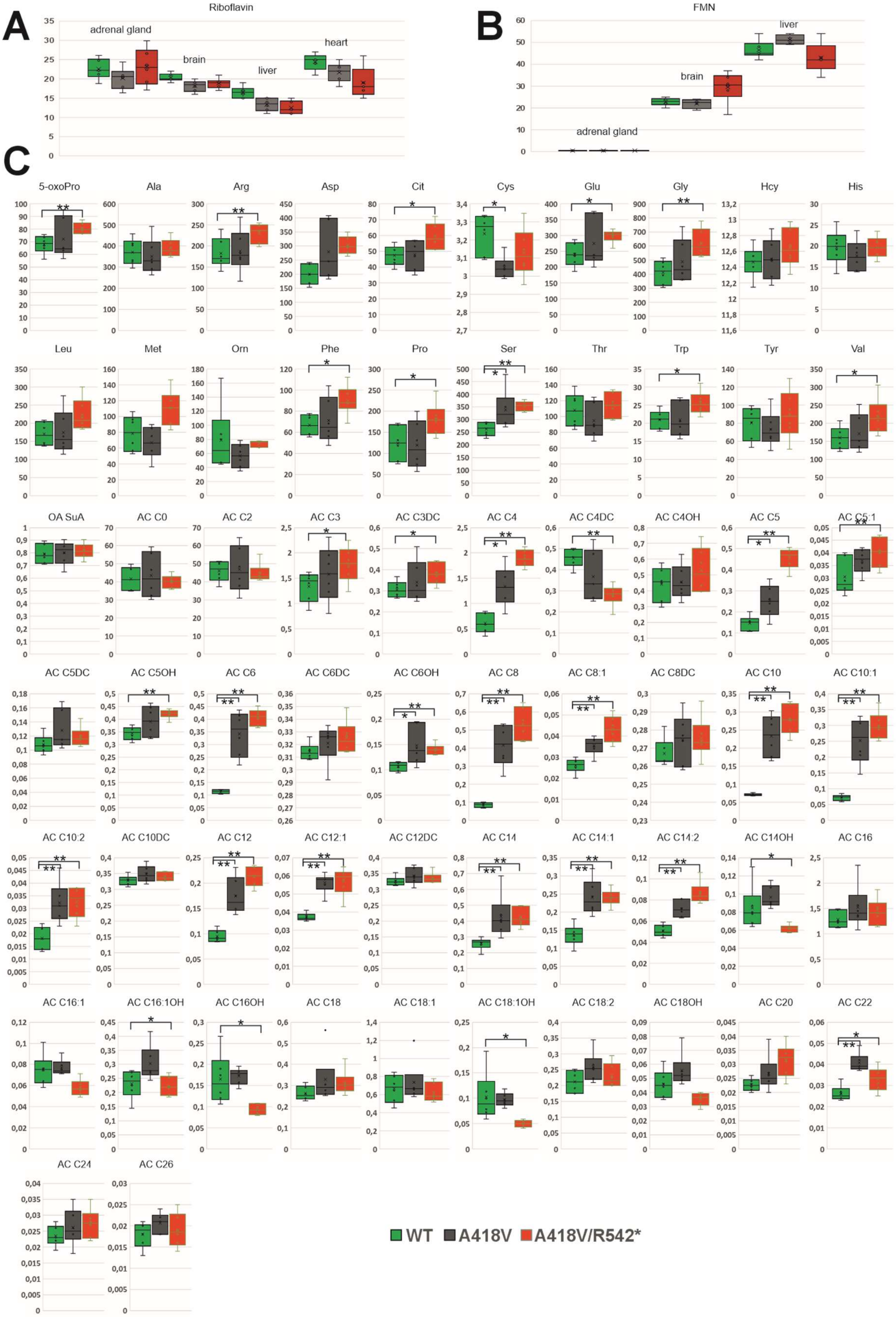

A
Riboflavin
adrenal gland
brain
liver
heart
B
FMN
adrenal gland
brain
liver
C
5-oxoPro
Ala
Arg
Asp
Cit
Cys
Glu
Gly
Hcy
His
Leu
Met
Orn
Phe
Pro
Ser
Thr
Trp
Tyr
Val
OA SuA
AC C0
AC C2
AC C3
AC C3DC
AC C4
AC C4DC
AC C4OH
AC C5
AC C5:1
AC C5DC
AC C5OH
AC C6
AC C6DC
AC C6OH
AC C8
AC C8:1
AC C8DC
AC C10
AC C10:1
AC C10:2
AC C10DC
AC C12
AC C12:1
AC C12DC
AC C14
AC C14:1
AC C14:2
AC C14OH
AC C16
AC C16:1
AC C16:1OH
AC C16OH
AC C18
AC C18:1
AC C18:1OH
AC C18:2
AC C18OH
AC C20
AC C22
AC C24
AC C26
WT
A418V
A418V/R542*

Figure S4. Metabolite quantity in mice with mutated *FLAD1*. (**A**) Riboflavin content in mouse adrenal gland, brain, liver, and heart extracts (tissue is indicated above the bars) WT (n = 6, 4Mo, male), A418V (n = 6, 4Mo, male), and A418V/R542* (n = 6, 4Mo, male), normalized by protein content (BCA), pmol/mg protein. The green color corresponds to the wild type, the gray color to *FLAD1*$^{A418V/A418V}$, and the red color to *FLAD1*$^{A418V/R542*}$ mice. (**B**) FMN content in mouse tissue extracts WT (n = 3-6, 4Mo, male), A418V (n = 6, 4Mo, male), and A418V/R542* (n = 2-6, 4Mo, male), normalized by protein content (BCA), pmol/mg protein. A total of 6 mice were analyzed per genotype, but in several samples, concentrations were below the detection limit. The green color corresponds to the wild type, the gray color to *FLAD1*$^{A418V/A418V}$, and the red color to *FLAD1*$^{A418V/R542*}$ mice. (**C**) Blood spot concentration of amino acids and acyl carnitines (indicated above the graphs). The color code and number of animals are the same as for panel (A). *$p < 0.05$, **$p < 0.01$ by Mann-Whitney U-test.

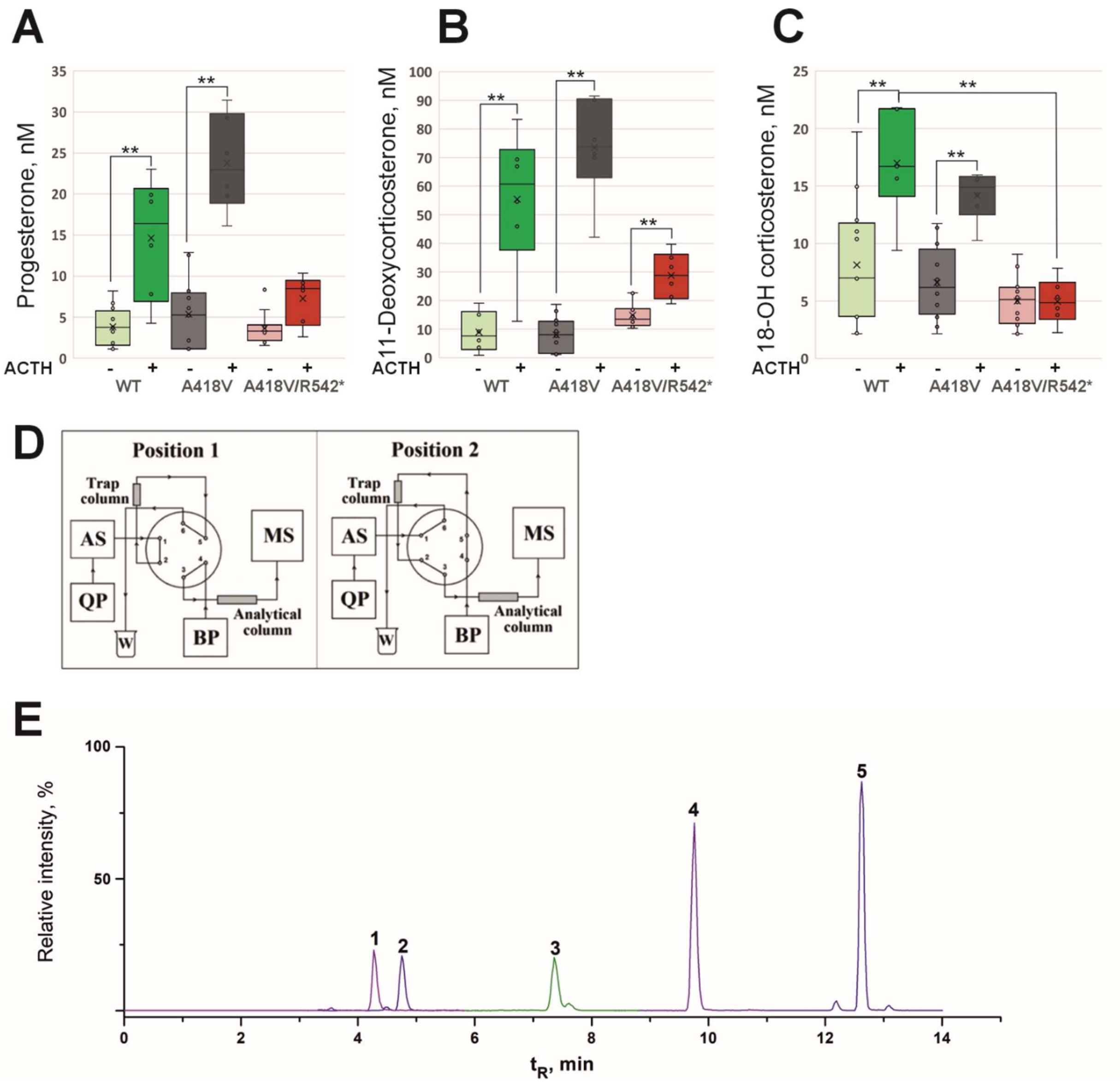

Figure S5. Analysis of steroids by HPLC-MS/MS (**A**) Progesterone concentration in blood plasma at steady state (pale colors, bars 1, 3, 5) and after ACTH (synacthen) stimulation (bright colors, bars 2, 4, 6), nM. Green color corresponds to the wild type (bars 1, 2, n = 12, 3Mo, male), gray color corresponds to *FLAD1*$^{A418V/A418V}$ (bars 3, 4, n = 6, 3Mo, male), while red color corresponds to the *FLAD1*$^{A418V/R542*}$ (bars 5, 6, n = 6, 3Mo, male) mice. *p < 0.05, **p < 0.01, ***p ≤ 0.001 by Mann-Whitney U-test. (**B**) 11-deoxycorticosterone concentration in blood plasma at steady state (pale colors, bars 1, 3, 5) and after ACTH (synacthen) stimulation (bright colors, bars 2, 4, 6), nM. Designation similar to that of panel (A). (**C**) 18-OH corticosterone concentration in blood plasma at steady state (pale colors, bars 1, 3, 5) and after ACTH (synacthen) stimulation (bright colors, bars 2, 4, 6), nM. Designation similar to that of panel (A). (**D**) Trap-and-elute 6-port 2-position valve configuration. MS – mass spectrometer, QP – quaternary pump, BP – binary pump, AS – autosampler, W – waste bottle. (**E**) Typical chromatogram of the steroid standards mixture (only quantifier MRM traces are shown). 1 – aldosterone, 2 – 18-hydroxycorticosterone, 3 – corticosterone, 4 – 11-deoxycorticosterone, 5 – progesterone.

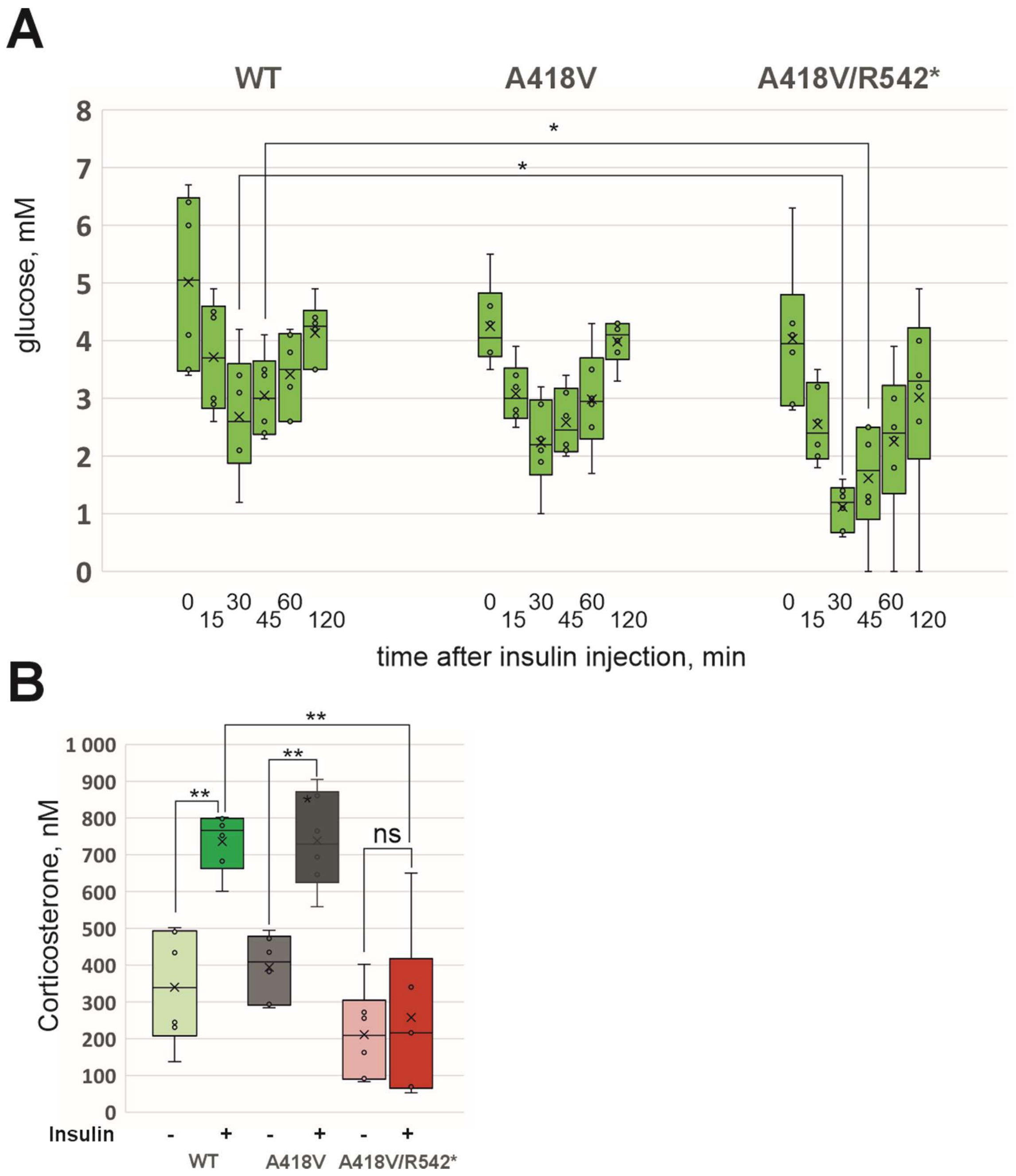

Figure S6. *FLAD1* mutations lead to age-dependent hypoglycemia. (A) Time course of the blood glucose concentration (mM) in mice following intraperitoneal injection of 0.75 mU/g of insulin per mouse weight after 4 hours of fasting. Time points 0, 15, 30, 45, 60, and 120 minutes are indicated below the graphs. Mice genotypes are shown above the graphs: wild type (left part, n = 6, male, 6 months), *FLAD1*$^{A418V/A418V}$ (central part, n = 6, male, 6 months), and *FLAD1*$^{A418V/R542*}$ (right part, n = 6, male, 6 months) mice. The experiment has been performed with the same 6-month-old mice, as they were analyzed at 3 months of age to obtain the data presented in Figure 5 (main text). (**B**) Corticosterone concentration in blood plasma at steady state (pale colors, bars 1, 3, 5) and after 0.75 mU/g insulin stimulation (bright colors, bars 2, 4, 6), nM. Green color corresponds to the wild type (bars 1, 2, n = 6, 3Mo, male), gray color corresponds to *FLAD1*$^{A418V/A418V}$ (bars 3, 4, n = 6, 3Mo, male), while red color corresponds to the *FLAD1*$^{A418V/R542*}$ (bars 5, 6, n = 6, 3Mo, male) mice. *$p < 0.05$, **$p < 0.01$ by Mann-Whitney U-test.

SUPPLEMENTARY REFERENCES

1. Kopanos C, et al. VarSome: the human genomic variant search engine. *Bioinformatics*. 2019;35(11):1978–1980.

2. Adzhubei I, Jordan DM, Sunyaev SR. Predicting functional effect of human missense mutations using PolyPhen-2. *Curr Protoc Hum Genet*. 2013;Chapter 7:Unit7.20.

3. Rogers MF, et al. FATHMM-XF: accurate prediction of pathogenic point mutations via extended features. *Bioinformatics*. 2018;34(3):511–513.

4. Taylor RW, et al. Use of Whole-Exome Sequencing to Determine the Genetic Basis of Multiple Mitochondrial Respiratory Chain Complex Deficiencies. *JAMA*. 2014;312(1):68.

5. Olsen RKJ, et al. Riboflavin-Responsive and -Non-responsive Mutations in FAD Synthase Cause Multiple Acyl-CoA Dehydrogenase and Combined Respiratory-Chain Deficiency. *The American Journal of Human Genetics*. 2016;98(6):1130–1145.

6. Yıldız Y, et al. Post-mortem detection of FLAD1 mutations in 2 Turkish siblings with hypotonia in early infancy. *Neuromuscular Disorders*. 2018;28(9):787–790.

7. Muru K, et al. *FLAD1* -associated multiple acyl-CoA dehydrogenase deficiency identified by newborn screening. *Molec Gen & Gen Med*. 2019;7(9):e915.

8. Hosseini SA, Shojaie L, Ghelichi-Ghojogh M. Detection of FLAD1 mutations and lipid storage myopathy in a 5-year-old boy: a case report study. *Annals of Medicine & Surgery*. 2023;85(5):2049–2051.

9. Auranen M, et al. Patient with multiple acyl-CoA dehydrogenation deficiency disease and FLAD1 mutations benefits from riboflavin therapy. *Neuromuscular Disorders*. 2017;27(6):581–584.

10. Ryder B, et al. A Novel Truncating FLAD1 Variant, Causing Multiple Acyl-CoA Dehydrogenase Deficiency (MADD) in an 8-Year-Old Boy. In: Morava E, et al., eds. *JIMD Reports, Volume 45*. Berlin, Heidelberg: Springer Berlin Heidelberg; 2018:37–44.

11. Lee YJ, et al. Infant with early onset bilateral facial and bulbar weakness: Successful treatment of riboflavin in multiple acyl-CoA dehydrogenase deficiency caused by biallelic nonsense FLAD1 variants. *Neuromuscular Disorders*. 2021;31(11):1194–1198.

12. Yamada K, et al. Flavin adenine dinucleotide synthase deficiency due to FLAD1 mutation presenting as multiple acyl-CoA dehydrogenation deficiency-like disease: A case report. *Brain and Development*. 2019;41(7):638–642.

13. Tolomeo M, et al. Retrograde response to mitochondrial dysfunctions associated to LOF variations in FLAD1 exon 2: unraveling the importance of RFVT2. *Free Radic Res*. 2022;56(7–8):511–525.

14. García-Villoria J, et al. *FLAD1* , encoding FAD synthase, is mutated in a patient with myopathy, scoliosis and cataracts. *Clinical Genetics*. 2018;94(6):592–593.

15. Vengalil S, et al. Mutation Spectrum of Primary Lipid Storage Myopathies. *Annals of Indian Academy of Neurology*. 2022;25(1):106–113.

16. Wen B, et al. A comparative study on riboflavin responsive multiple acyl-CoA dehydrogenation deficiency due to variants in FLAD1 and ETFDH gene. *J Hum Genet*. 2024;69(3–4):125–131.
