# Supplementary material for: A familial case of FLAD1 protein deficiency associated with impaired adrenal steroidogenesis
Source: JCI Insight. 2026 Sep 8;11(17):e198799. doi: 10.1172/jci.insight.198799 (PMC13564092; doi:10.1172/jci.insight.198799)
Supplement: Supplementary file 2 [file jciinsight-11-198799-s204.pdf]

Full unedited gel for Figure 3, E

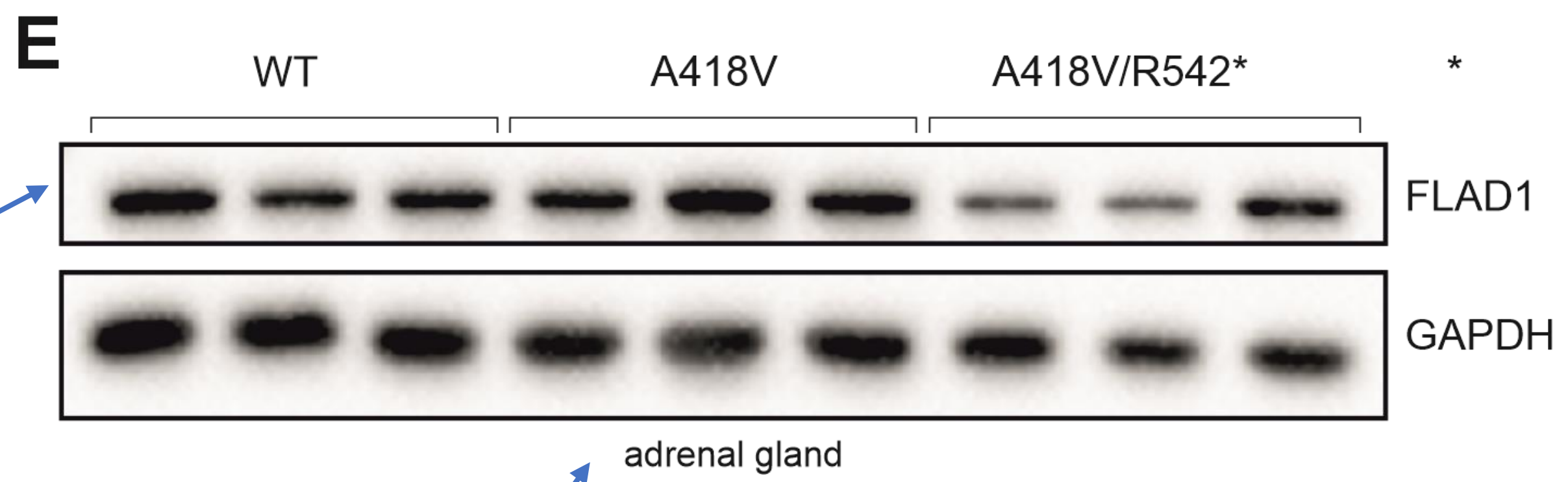

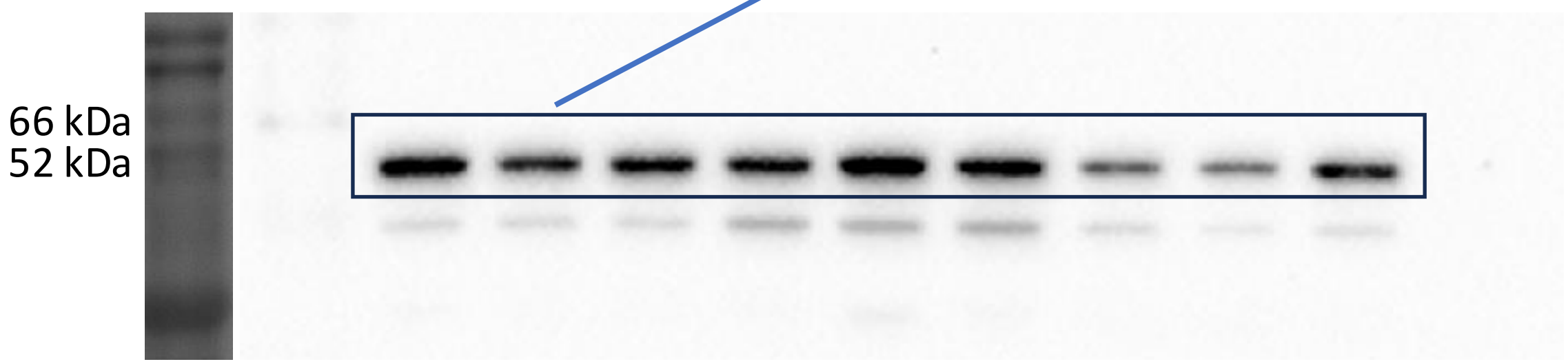

Anti-Flad antibody, PA5-89731

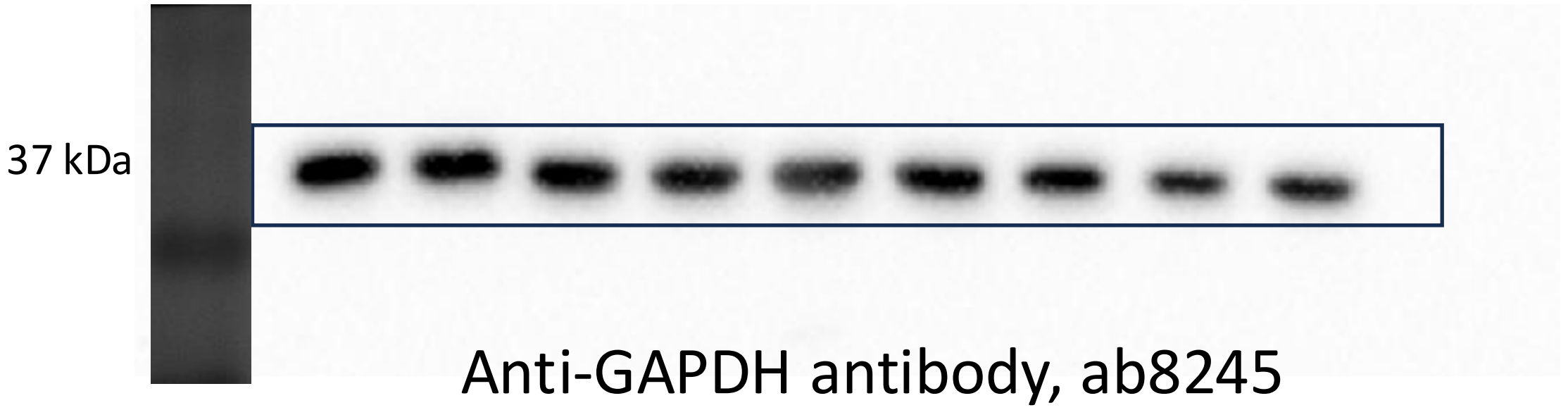

Anti-GAPDH antibody, ab8245

Protein marker
BiolabMix Rav-10, PS-1050

Full unedited gel for Figure S3, A

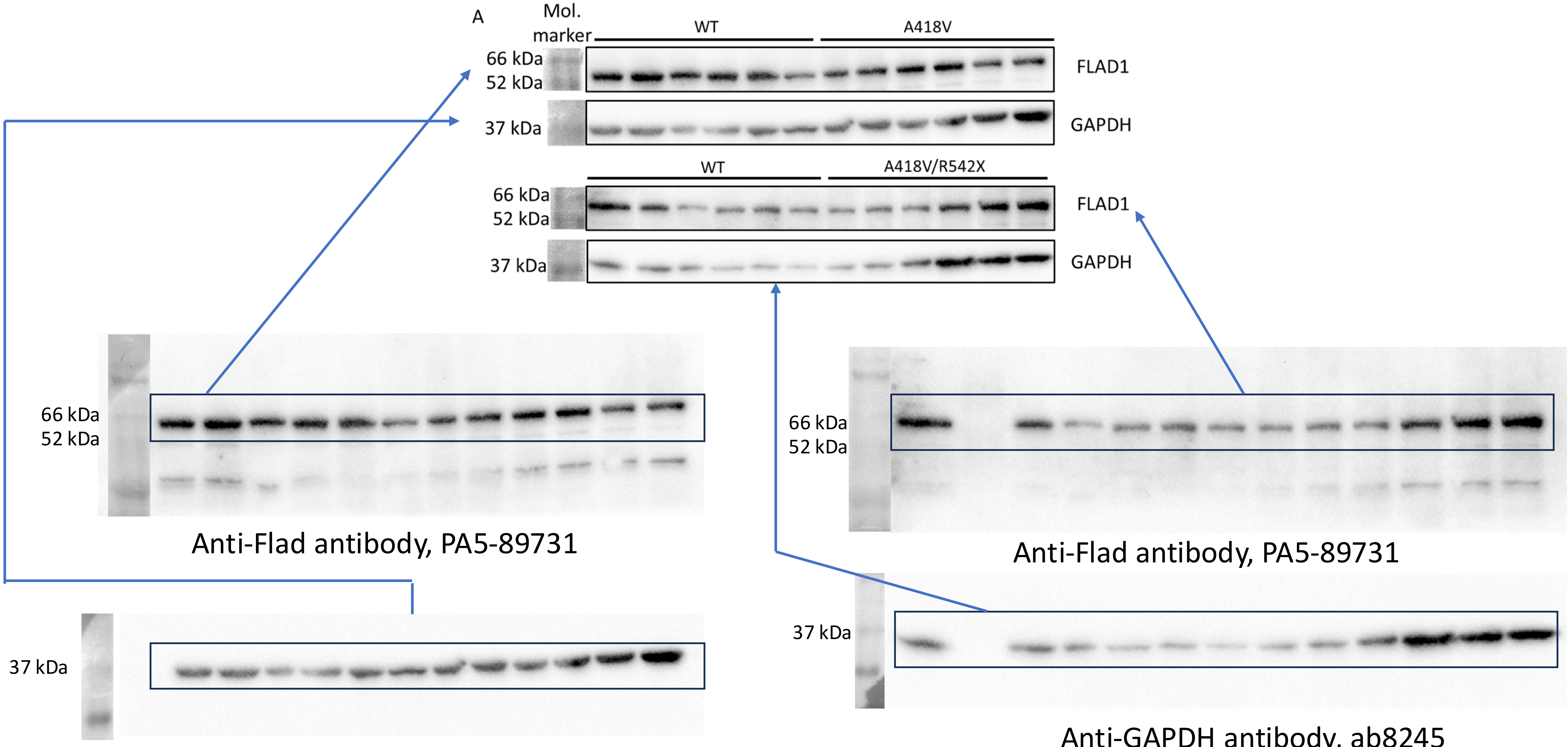

Anti-GAPDH antibody, ab8245

Protein marker
BiolabMix Rav-10, PS-1050

Full unedited gel for Figure S3, B

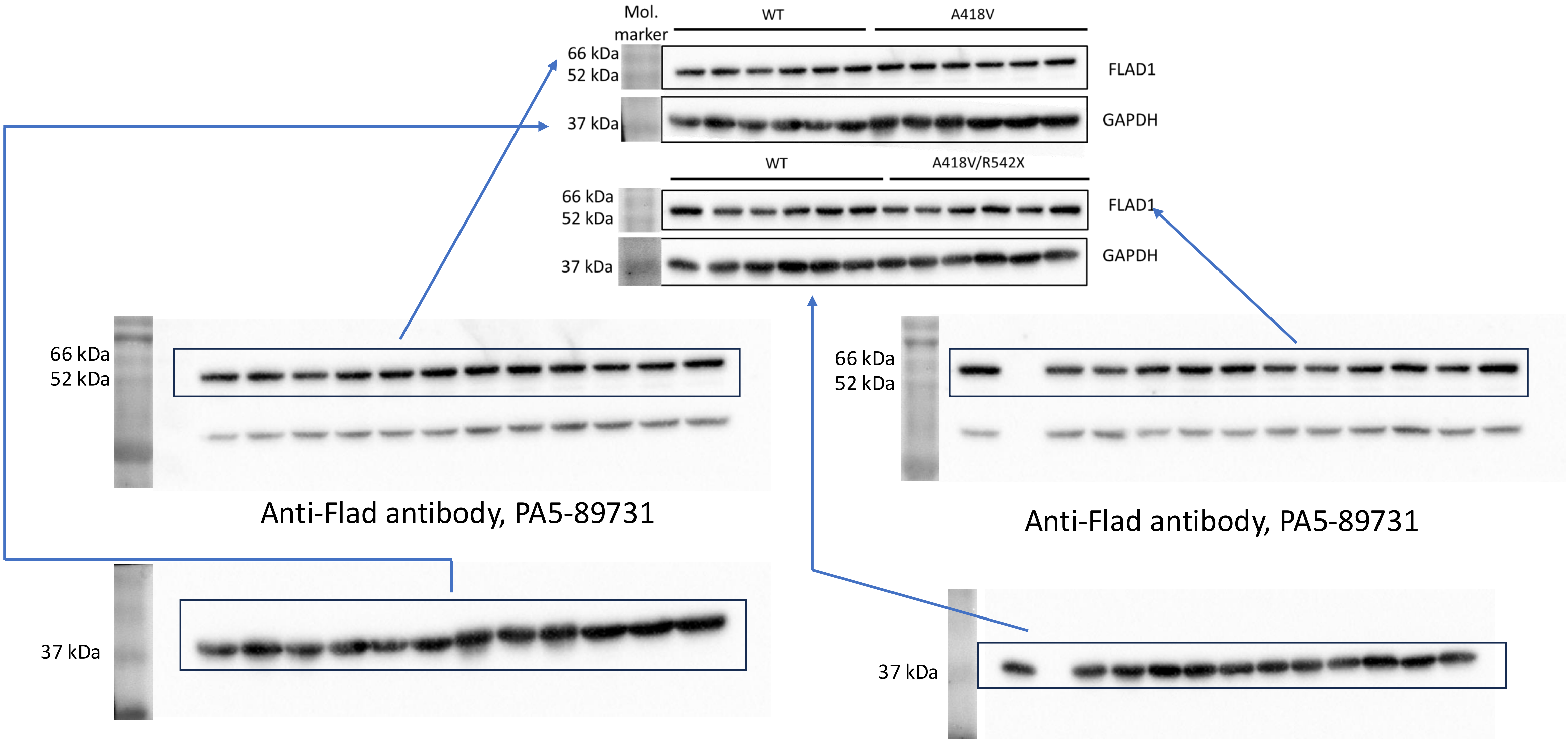

Anti-Flad antibody, PA5-89731

Anti-Flad antibody, PA5-89731

Anti-GAPDH antibody, ab8245

Anti-GAPDH antibody, ab8245

Protein marker
BiolabMix Rav-10, PS-1050

Full unedited gel for Figure S3, C

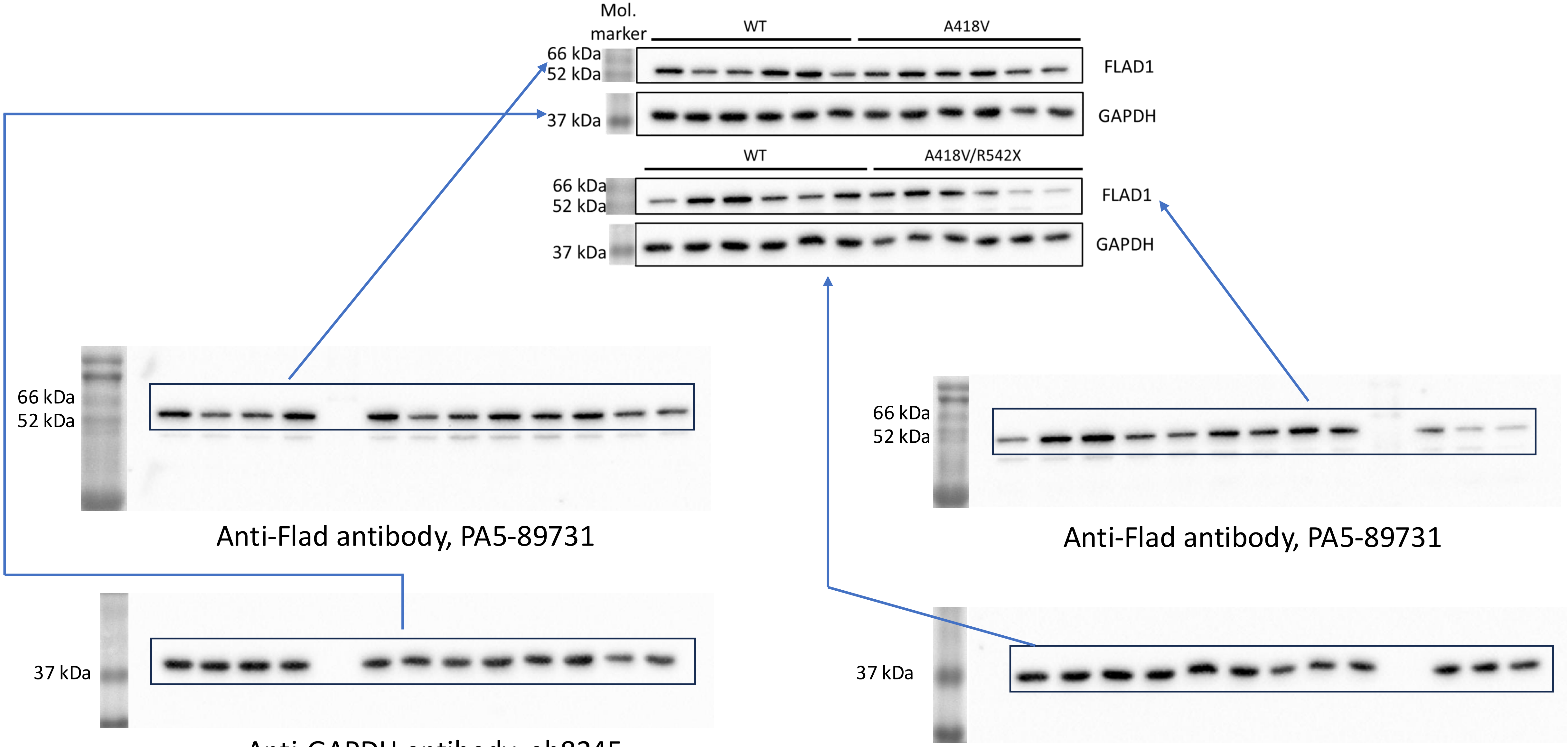

Protein marker
BiolabMix Rav-10, PS-1050
